# Supplementary material for: PDn-3 DPA Pathway Regulates Human Monocyte Differentiation and Macrophage Function
Source: Cell Chem Biol. 2018 Jun 21;25(6):749–760.e9. doi: 10.1016/j.chembiol.2018.04.017 (PMC6024030; doi:10.1016/j.chembiol.2018.04.017)
Supplement: Document S1. Figures S1–S5 and Tables S1–S3 [file mmc1.pdf]

Cell Chemical Biology, Volume *25*

Supplemental Information

$PD_{n\text{-}3\ DPA}$ Pathway Regulates Human Monocyte Differentiation and Macrophage Function

Kimberly Pistorius, Patricia R. Souza, Roberta De Matteis, Shani Austin-Williams, Karoline G. Primdahl, Anders Vik, Francesca Mazzacuva, Romain A. Colas, Raquel M. Marques, Trond V. Hansen, and Jesmond Dalli

Figure S1

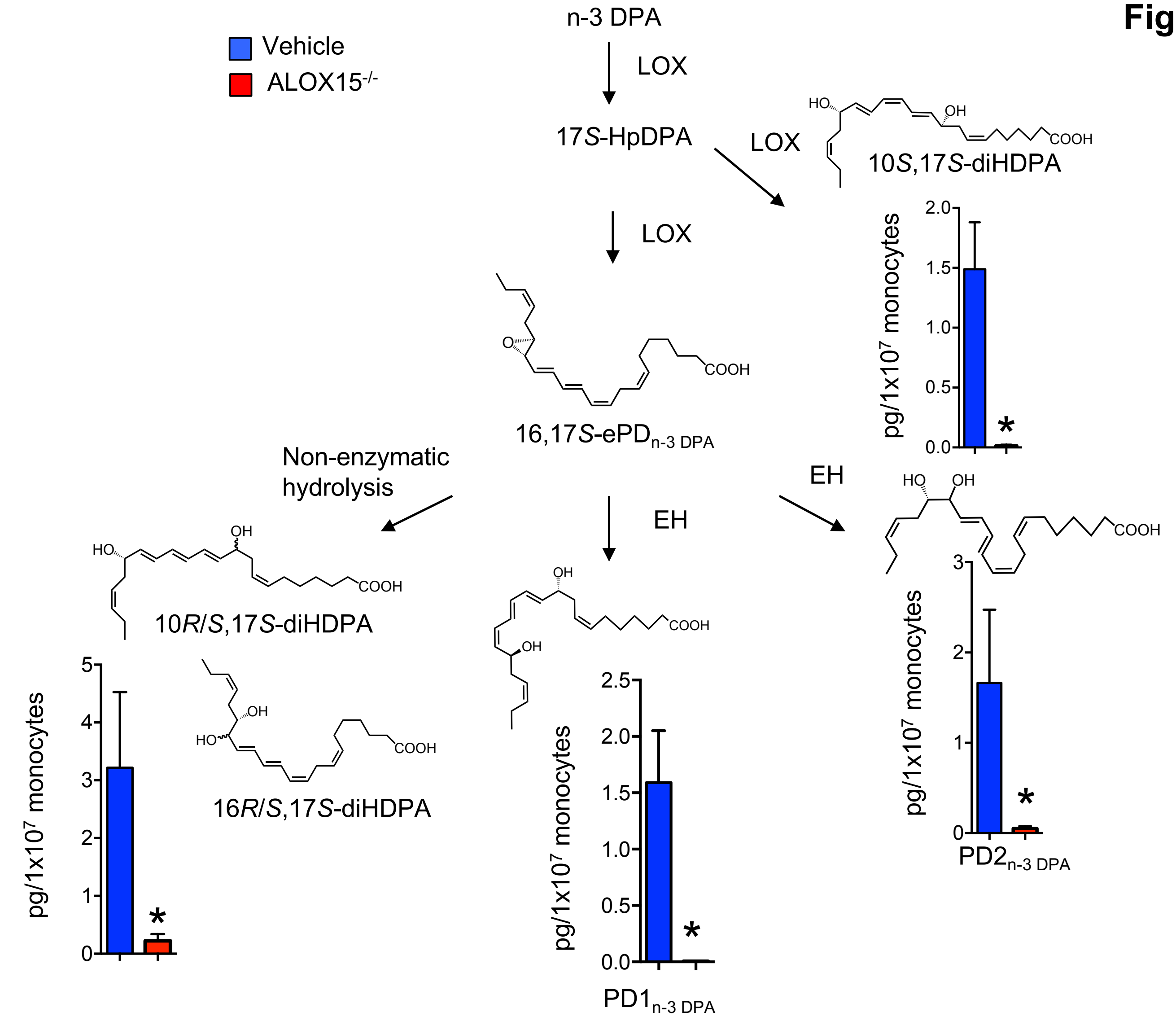

Vehicle
ALOX15-/-
n-3 DPA
LOX
17S-HpDPA
LOX
10S,17S-diHDPA
LOX
16,17S-ePDn-3 DPA
Non-enzymatic hydrolysis
EH
EH
10R/S,17S-diHDPA
16R/S,17S-diHDPA
PD1n-3 DPA
PD2n-3 DPA
pg/1x10^7 monocytes
COOH
HO
OH
*

Figure S2

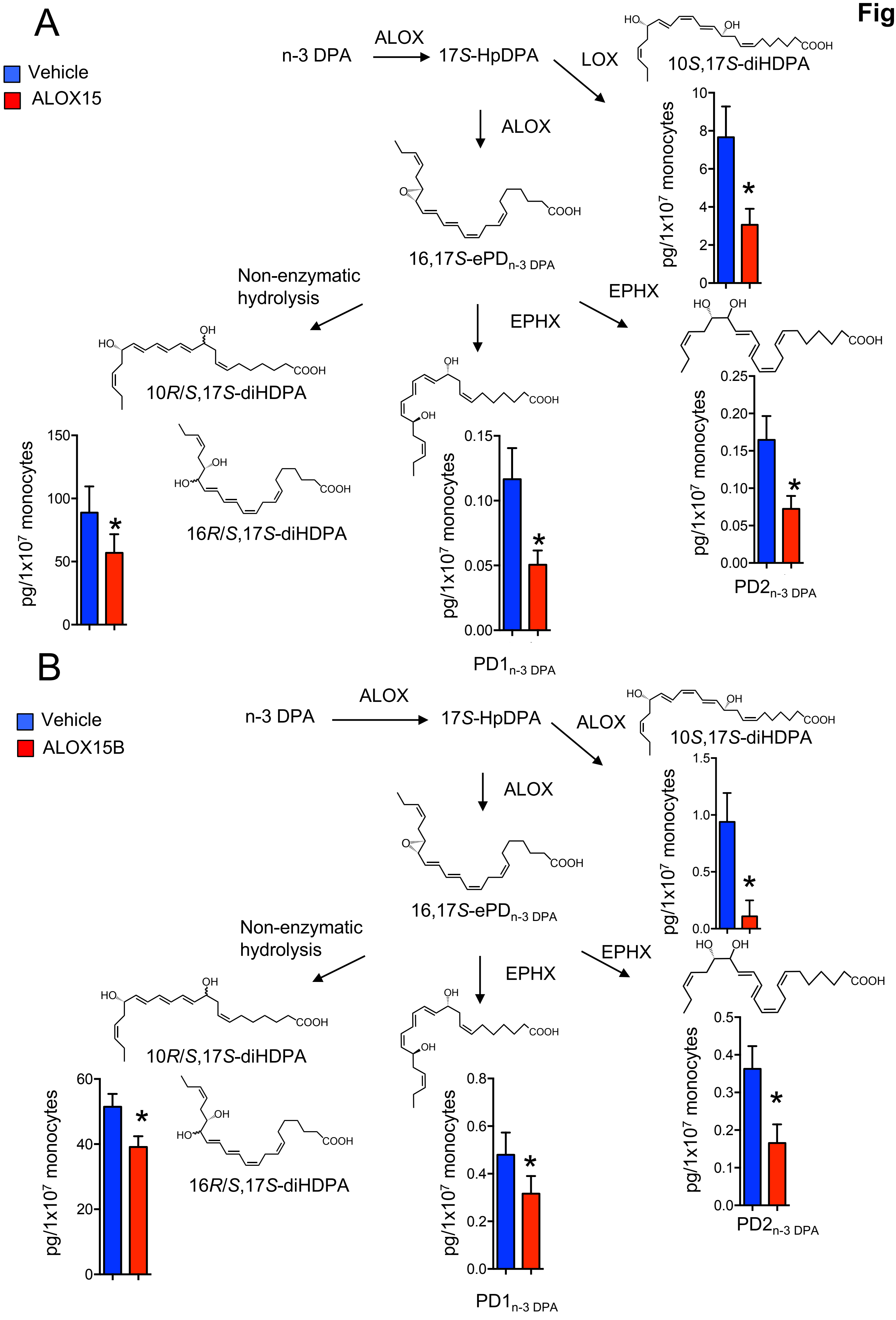
A
Vehicle
ALOX15
n-3 DPA
ALOX
17S-HpDPA
LOX
10S,17S-diHDPA
ALOX
16,17S-ePD n-3 DPA
Non-enzymatic hydrolysis
EPHX
EPHX
10R/S,17S-diHDPA
16R/S,17S-diHDPA
PD1 n-3 DPA
PD2 n-3 DPA
pg/1x10^7 monocytes
B
Vehicle
ALOX15B
n-3 DPA
ALOX
17S-HpDPA
ALOX
10S,17S-diHDPA
ALOX
16,17S-ePD n-3 DPA
Non-enzymatic hydrolysis
EPHX
EPHX
10R/S,17S-diHDPA
16R/S,17S-diHDPA
PD1 n-3 DPA
PD2 n-3 DPA
pg/1x10^7 monocytes

Figure S3

A

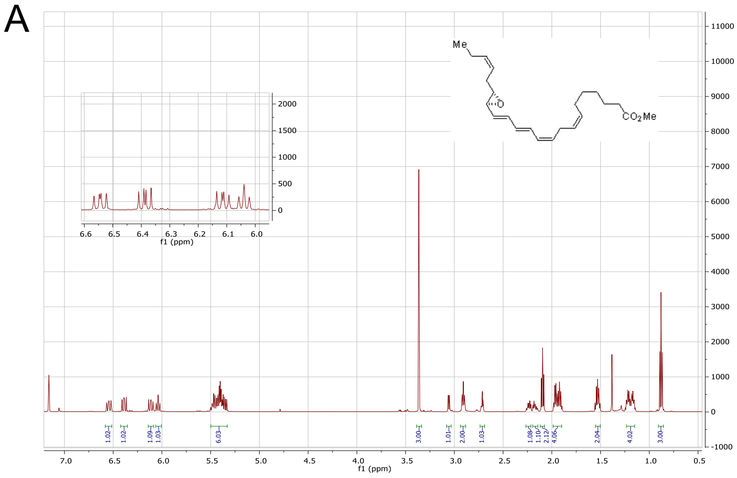

B

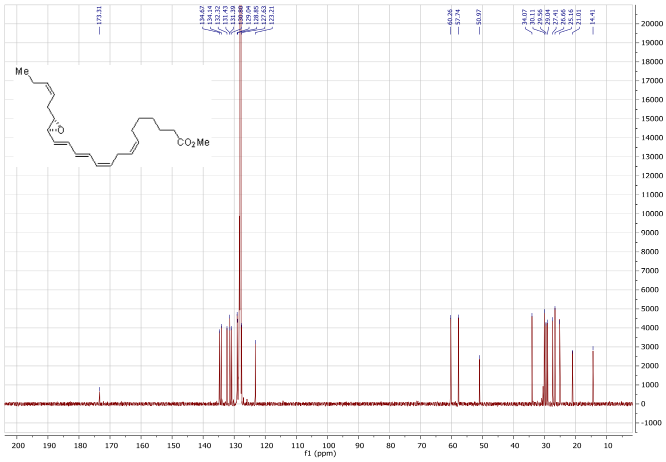

C

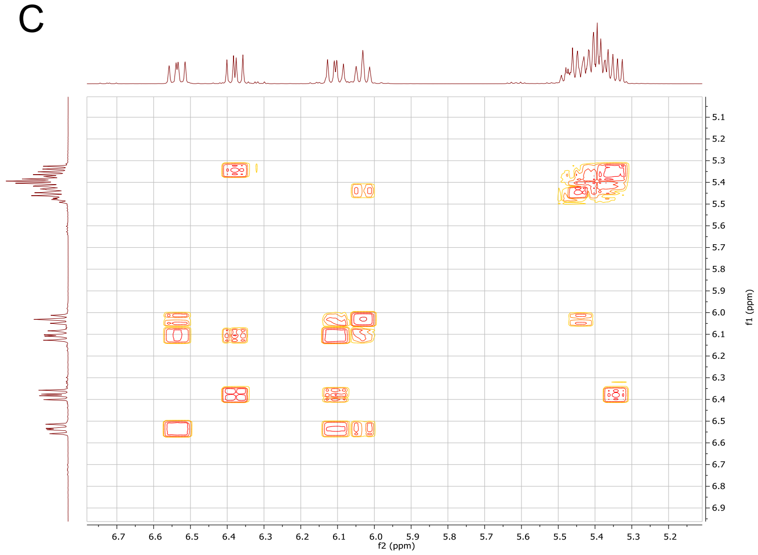

D

Elemental Analysis Report

**Analysis Info**		Acquisition Date	10/20/2016 10:56:29 AM
Sample Name		Analysis Name	D:\Data\maxis2016\12053.d
Method	ESI_pos_50_1500.m		

Acquisition Parameter

Source Type	ESI	Ion Polarity	Positive	Set Nebulizer	0.4 Bar
Focus	Not active	Set Capillary	3500 V	Set Dry Heater	200 °C
Scan Begin	50 m/z	Set End Plate Offset	-500 V	Set Dry Gas	4.0 l/min
Scan End	1500 m/z	Set Charging Voltage	2000 V	Set Divert Valve	Waste
		Set Corona	0 nA	Set APCI Heater	0 °C

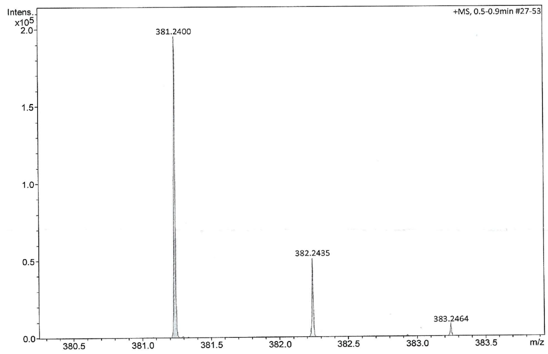

Meas. m/z	Ion Formula	m/z	err [ppm]
381.2400	C23H34NaO3	381.2400	-0.1
	C21H29N6O	381.2397	-0.8
	C20H33N2O5	381.2384	-4.3

Figure S4

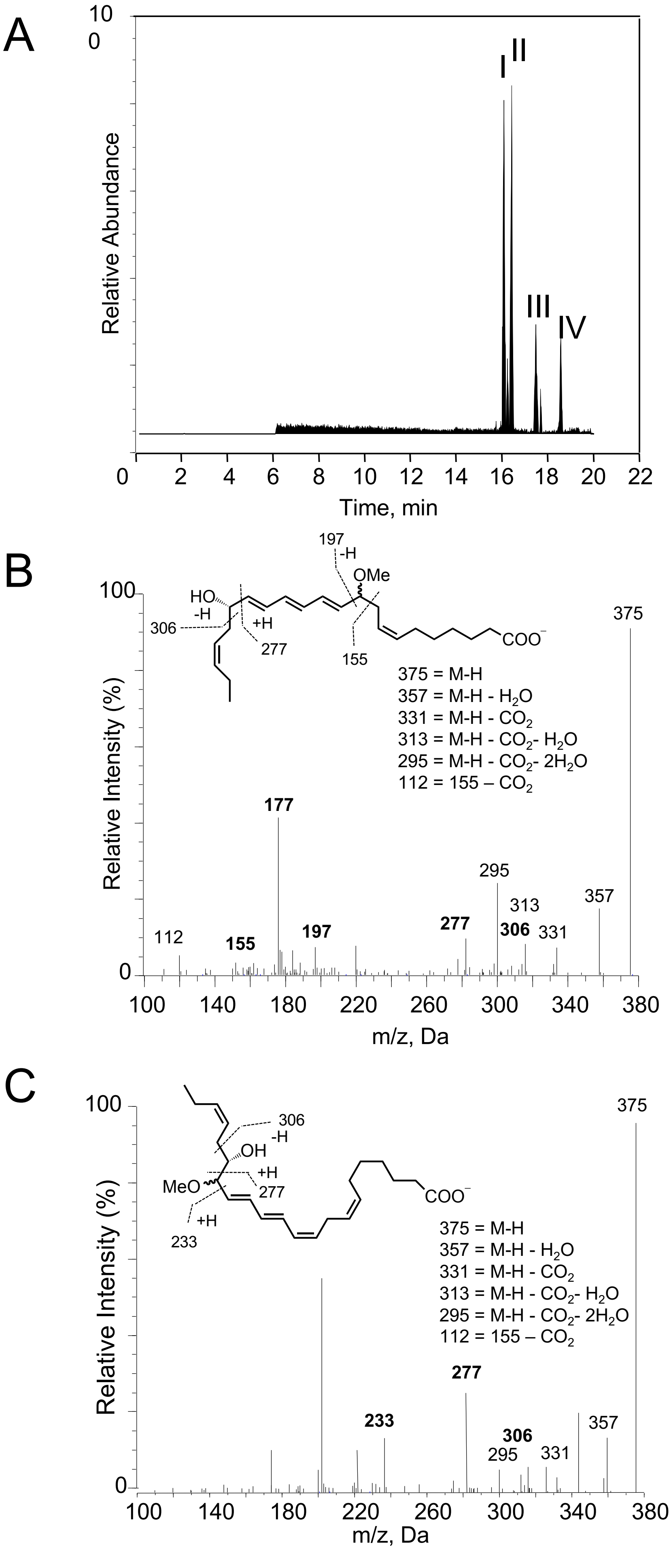
A
100
Relative Abundance
I
II
III
IV
0 2 4 6 8 10 12 14 16 18 20 22
Time, min
B
197
-H
OMe
HO
-H
306
+H
277
155
COO⁻
100
375
375 = M-H
357 = M-H - H_2O
331 = M-H - CO_2
313 = M-H - CO_2- H_2O
295 = M-H - CO_2- $2H_2O$
112 = 155 – CO_2
Relative Intensity (%)
177
295
313
357
112
155
197
277
306
331
0
100 140 180 220 260 300 340 380
m/z, Da
C
100
306
-H
OH
+H
277
MeO
+H
233
COO⁻
375
375 = M-H
357 = M-H - H_2O
331 = M-H - CO_2
313 = M-H - CO_2- H_2O
295 = M-H - CO_2- $2H_2O$
112 = 155 – CO_2
Relative Intensity (%)
277
233
306
357
295
331
0
100 140 180 220 260 300 340 380
m/z, Da

Figure S5

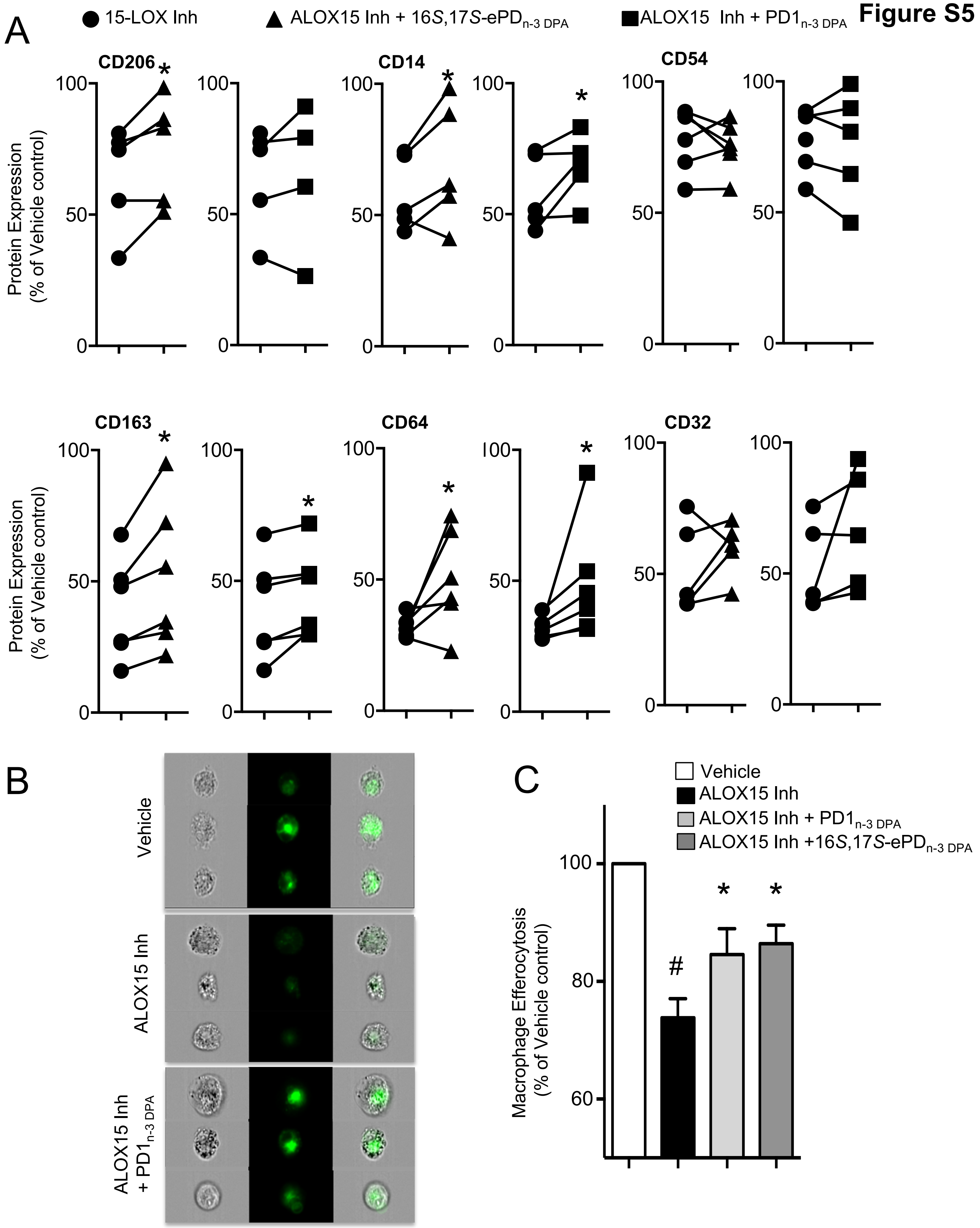

A
15-LOX Inh
ALOX15 Inh + 16S,17S-ePD n-3 DPA
ALOX15 Inh + PD1 n-3 DPA
CD206
CD14
CD54
CD163
CD64
CD32
Protein Expression
(% of Vehicle control)
100
50
0
B
Vehicle
ALOX15 Inh
ALOX15 Inh
+ PD1 n-3 DPA
C
Vehicle
ALOX15 Inh
ALOX15 Inh + PD1 n-3 DPA
ALOX15 Inh +16S,17S-ePD n-3 DPA
Macrophage Efferocytosis
(% of Vehicle control)
100
80
60
#
*

Supplemental Figures

Figure S1: Decreased $PD_{n\text{-}3\ DPA}$ in ALOX15 deficient mice. Related to Figure 1 and Table S1.

Bone marrow monocytes were isolated from WT or ALOX15 deficient mice. These ($2x10^6$ cells/ml) were then incubated with *E. coli* ($1x10^8$ CFU/mL) for 30 min (37°C; $PBS^{+/+}$). Incubations were quenched with ice-cold methanol and products identified and quantified using lipid mediator profiling. Results are mean ± SEM. n = 4 mice per group. * P<0.05.

Figure S2: Knockdown of both ALOX15 and ALOX15B reduces $PD_{n\text{-}3\ DPA}$ biosynthesis in human monocytes. Related to Figure 1.

Human monocytes were transfected with shRNA to (A) ALOX15, (B) ALOX15B or CT shRNA (see methods for details). The cells ($1x10^6$ cells/mL) were incubated with *E. coli* ($5x10^7$ CFU/mL) for 45 min (37°C) and $PD_{n\text{-}3\ DPA}$ levels were ascertained using lipid mediator profiling. Results are mean ± SEM. n = 5 donors.

Figure S3: Characterizing the synthetic 16*S*,17*S*-$ePD_{n\text{-}3\ DPA}$. Related to Figure 3. (A) ^{1}H-NMR spectrum of methyl (7*Z*,10*Z*,12*E*,14*E*)-15-((2*S*,3*S*)-3-((*Z*)-pent-2-en-1-yl)oxiran-2-yl)pentadeca-7,10,12,14-tetraenoate (methyl $ePD1_{n\text{-}3\ DPA}$). (B) ^{13}C-NMR spectrum of methyl (7*Z*,10*Z*,12*E*,14*E*)-15-((2*S*,3*S*)-3-((*Z*)-pent-2-en-1-yl)oxiran-2-yl)pentadeca-7,10,12,14-tetraenoate (methyl $ePD1_{n\text{-}3\ DPA}$). (C) HSQC spectrum of methyl (7*Z*,10*Z*,12*E*,14*E*)-15-((2*S*,3*S*)-3-((*Z*)-pent-2-en-1-yl)oxiran-2-yl)pentadeca-7,10,12,14-tetraenoate (methyl $ePD1_{n\text{-}3\ DPA}$). (D) HRMS of methyl (7*Z*,10*Z*,12*E*,14*E*)-15-((2*S*,3*S*)-3-((*Z*)-pent-2-en-1-yl)oxiran-2-yl)pentadeca-7,10,12,14-tetraenoate (methyl $ePD1_{n\text{-}3\ DPA}$).

Figure S4: Acid alcohol trapping product profile for synthetic 16*S*,17*S*-$ePD_{n\text{-}3\ DPA}$. Related to Figure 2.

The epoxide was incubated in acidified methanol and products were profiled using LC/MS-MS. (A) MRM chromatogram for ion pairs *m/z* 375>277. (B-C) MS-MS spectra employed in the identification of (B) 10-methoxy,17*S*-hydroxy-7*Z*,11*E*,13*E*,15*E*,19Z-docosapentaenoic acid and (C) 16-methoxy,17S-hydroxy-7Z,10Z,12E,14E,19Z-docosapentaenoic acid in monocyte incubations.

Figure S5: 16*S*,17*S*-$ePD_{n\text{-}3\ DPA}$ and $PD1_{n\text{-}3\ DPA}$ rectify monocyte differentiation following ALOX15 inhibition restoring macrophage responses. Related to Figure 7.

Human monocytes were incubated with M-CSF (20ng/mL) and 15-LOX inhibitor (10μM), ALOX15 inhibitor + 16*S*, 17*S*-$ePD_{n\text{-}3\ DPA}$ (1nM), ALOX15 inhibitor + $PD1_{n\text{-}3\ DPA}$ (1nM), or vehicle (37°C, 5% CO_2 in RPMI supplemented with human serum). On day 7 (A) cells were collected and expression of lineage markers was determined using fluorescently labelled antibodies and flow cytometry. (B-C) cells were incubated with fluorescently labelled apoptotic cells for 45 min (37°C) and phagocytosis was evaluated using (B) ImageStream (C) fluorescent plate reader . * P < 0.05 vs ALOX15 inhibitor incubations; # P < 0.05 versus vehicle incubations.

Supplemental Tables

Table S1: ALOX15 inhibitor shifts human monocyte-derived macrophage lipid mediator profiles Related to Figure 1.

**DHA bioactive metabolome**	**Q1**	**Q3**	**Vehicle**	**ALOX15 inhibitor**
RvD1	375	141	26.8 ± 4.6	13.3 ± 3.3 *
RvD2	375	141	127.6 ± 42.8	62.9 ± 42.5 *
RvD3	375	147	19.3 ± 7.6	10.4 ± 5.2
RvD4	375	101	172.6 ± 33.9	127.7 ± 42.7
RvD5	359	199	104.1 ± 37.4	65.6 ± 31.9 *
RvD6	359	101	639.4 ± 233.8	532.5 ± 187.5 *
17R-RvD1	375	141	47.0 ± 18.8	19.0 ± 5.7
17R-RvD3	375	147	12.5 ± 4.2	8.3 ± 4.3
PD1	359	153	24.4 ± 6.2	16.6 ± 5.7 *
10S,17S-diHDHA	359	153	309.0 ± 114.3	220.8 ± 92.3 *
17R-PD1	359	153	18.7 ± 10.7	17.0 ± 12.3
22-OH-PD1	375	153	7.4 ± 3.8	4.8 ± 3.1 *
MaR1	359	221	64.2 ± 56.6	22.1 ± 10.5
MaR2	359	191	184.0 ± 98.9	161.2 ± 88.0
7S,14S-diHDHA	359	221	259.9 ± 87.3	427.0 ± 143.6
22-OH-MaR1	375	221	196.1 ± 79.5	200.1 ± 74.6
4S,14S-diHDHA	359	101	69.7 ± 61.7	20.5 ± 9.9
14-oxo-MaR1	357	248	16.3 ± 7.4	9.5 ± 5.4
**n-3 DPA bioactive metabolome**				
RvT1	377	239	39.9 ± 21.2	26.7 ± 12.3
RvT2	377	197	34.6 ± 21.6	25.8 ± 16.7
RvT3	377	197	22.1 ± 12.8	10.0 ± 3.9
RvT4	361	211	36.0 ± 11.2	62.7 ± 34.6
$RvD1_{n\text{-}3\ DPA}$	377	143	39.8 ± 16.5	28.0 ± 15.0 *
$RvD2_{n\text{-}3\ DPA}$	377	143	21.2 ± 8.5	12.9 ± 3.1
$RvD5_{n\text{-}3\ DPA}$	361	199	125.2 ± 32.7	76.6 ± 16.1 *
$PD1_{n\text{-}3\ DPA}$	361	183	32.5 ± 9.1	11.0 ± 1.2 *
$PD2_{n\text{-}3\ DPA}$	361	263	27.5 ± 10.0	13.4 ± 8.2 *
10S, 17S-diHDPA	361	183	33.9 ± 7.6	19.5 ± 3.6 *
Δ15trans-$PD1_{n\text{-}3\ DPA}$	361	183	61.0 ± 25.9	52.2 ± 23.4 *
10epi-Δ15trans-$PD1_{n\text{-}3\ DPA}$	361	183	75.3 ± 19.4	55.0 ± 20.8 *
$MaR1_{n\text{-}3\ DPA}$	361	249	13.8 ± 7.3	18.2 ± 11.4
7S, 14S-diHDPA	361	249	77.9 ± 28.2	47.6 ± 14.2
**EPA bioactive metabolome**				
RvE1	349	195	18.5 ± 11.9	18.6 ± 12.7
RvE2	333	199	269.5 ± 102.2	246.0 ± 112.1
RvE3	333	201	93.8 ± 33.6	90.7 ± 39.9
**AA bioactive metabolome**				
LXA_4	351	217	514.2 ± 307.5	422.7 ± 239.5
LXB_4	351	221	602.9 ± 280.3	492.4 ± 228.9 *
5S,15S-diHETE	335	235	5259.1 ± 1992.8	3585.6 ± 1638.2 *
15R-LXA_4	351	217	386.2 ± 172.4	308.7 ± 146.9
15R-LXB_4	351	221	1848.8 ± 855.8	1283.9 ± 516.3
LTB_4	335	195	175.8 ± 170.4	60.1 ± 58.7
5S,12S-diHETE	335	195	1145.4 ± 596.4	1073.1 ± 586.4
20-OH-LTB_4	351	195	1.9 ± 0.6	1.4 ± 0.5
PGD_2	351	189	2191.3 ± 326.6	1277.4 ± 251.7 *
PGE_2	351	189	1983.8 ± 901.7	1151.9 ± 507.9
$PGF_{2\alpha}$	353	193	466.7 ± 196.9	230.1 ± 45.7
TxB_2	369	169	63661.3 ± 43046.4	19094.8 ± 14290.9

Human monocytes were incubated with M-CSF (20ng/ml) and either a ALOX15 inhibitor or vehicle (37^oC, 5% CO_2). On day 7 incubations were quenched with ice-cold methanol and lipid mediators identified using LC/MS-MS based profiling. Results are mean ± SEM and expressed in pg/1x10^7 cells. Q1, M-H (parent ion); and Q3, diagnostic ion in the MS-MS (daughter ion). * $p<0.05$ using Mann Whitney test.

Table S2: Altered lipid mediator profiles in monocytes from ALOX15 deficient mice. Related to Figure 1 and Supplemental Figure 1.

**DHA bioactive metabolome**	**Q1**	**Q3**	**WT**	**ALOX15$^{-/-}$**
RvD1	375	141	1.63 ± 0.78	0.19 ± 0.20
RvD2	375	141	2.00 ± 1.37	0.20 ± 0.17
RvD3	375	147	0.17 ± 0.08	0.01 ± 0.02
RvD4	375	101	1.96 ± 1.31	0.14 ± 0.07
RvD5	359	199	23.03 ± 11.87	2.99 ± 2.14
RvD6	359	101	0.60 ± 0.34	0.00 ± 0.00
17R-RvD1	375	141	0.83 ± 0.30	0.08 ± 0.08 *
17R-RvD3	375	147	0.66 ± 0.34	0.10 ± 0.06
PD1	359	153	2.08 ± 1.15	0.13 ± 0.07
10S,17S-diHDHA	359	153	31.28 ± 17.71	0.30 ± 0.19
17R-PD1	359	153	1.69 ± 1.17	0.02 ± 0.01
22-OH-PD1	375	153	0.16 ± 0.11	0.05 ± 0.04
MaR1	359	221	17.48 ± 4.89	0.57 ± 0.62 *
MaR2	359	191	1.55 ± 1.17	0.07 ± 0.07
7S,14S-diHDHA	359	221	8.52 ± 4.03	0.14 ± 0.12 *
22-OH-MaR1	375	221	2.70 ± 2.40	0.32 ± 0.32
4S,14S-diHDHA	359	101	3.22 ± 2.30	0.15 ± 0.14
14-oxo-MaR1	357	248	1.20 ± 0.95	0.11 ± 0.10
**n-3 DPA bioactive metabolome**				
RvT1	377	239	15.45 ± 4.39	1.40 ± 1.46 *
RvT2	377	197	9.08 ± 4.20	0.97 ± 0.43 *
RvT3	377	197	2.93 ± 1.67	0.11 ± 0.07
RvT4	361	211	34.31 ± 19.67	0.91 ± 1.03
RvD1$_{n\text{-}3\ DPA}$	377	143	2.48 ± 2.06	0.33 ± 0.20
RvD2$_{n\text{-}3\ DPA}$	377	143	0.97 ± 0.52	0.15 ± 0.09
RvD5$_{n\text{-}3\ DPA}$	361	199	3.67 ± 0.13	1.08 ± 0.23 *
PD1$_{n\text{-}3\ DPA}$	361	183	1.59 ± 0.53	0.00 ± 0.00 *
PD2$_{n\text{-}3\ DPA}$	361	263	1.86 ± 0.86	0.05 ± 0.03 *
10S, 17S-diHDPA	361	183	1.49 ± 0.45	0.01 ± 0.01 *
Δ15trans-PD1$_{n\text{-}3\ DPA}$	361	183	1.93 ± 0.99	0.08 ± 0.04 *
10epi-Δ15trans-PD1$_{n\text{-}3\ DPA}$	361	183	1.49 ± 0.45	0.14 ± 0.10 *
MaR1$_{n\text{-}3\ DPA}$	361	249	0.70 ± 0.48	0.08 ± 0.08
7S, 14S-diHDPA	361	249	8.85 ± 5.22	0.57 ± 0.43
**EPA bioactive metabolome**				
RvE1	349	195	2.36 ± 1.43	0.28 ± 0.24
RvE2	333	199	1.39 ± 0.95	0.25 ± 0.15
RvE3	333	201	3.11 ± 1.88	0.42 ± 0.42
**AA bioactive metabolome**				
LXA$_4$	351	217	1.66 ± 0.69	0.03 ± 0.02 *
LXB$_4$	351	221	12.41 ± 4.13	4.86 ± 3.35
5S,15S-diHETE	335	235	84.99 ± 58.58	6.92 ± 4.78
15R-LXA$_4$	351	217	2.02 ± 0.83	6.03 ± 4.01
15R-LXB$_4$	351	221	1.92 ± 1.12	0.28 ± 0.14
LTB$_4$	335	195	348.45 ± 98.93	97.71 ± 83.25 *
5S,12S-diHETE	335	195	0.00 ± 0.00	0.00 ± 0.00
20-OH-LTB$_4$	351	195	2.73 ± 1.81	0.02 ± 0.02
PGD$_2$	351	189	1256.77 ± 411.18	129.81 ± 104.81 *
PGE$_2$	351	189	397.88 ± 215.48	32.80 ± 26.30
PGF$_{2\alpha}$	353	193	730.81 ± 330.29	71.06 ± 65.35 *
TxB$_2$	369	169	2374.02 ± 784.55	131.47 ± 112.91 *

Bone marrow monocytes (2x10^6 cells/mL) were incubated with *E. coli* (2x10^8 cells/mL) for 45 minutes at 37°C. Incubations were quenched with ice-cold methanol and lipid mediators identified using LC/MS-MS based profiling. Results are mean ± SEM and expressed in pg/1x10^7 cells. Q1, M-H (parent ion); and Q3, diagnostic ion in the MS-MS (daughter ion). * $p<0.05$ using Mann Whitney test.

Table S3: Tissue resident macrophages from ALOX15 mice display and altered expression of lineage markers that is rescued by $PD1_{n\text{-}3\ DPA}$ administration. Related to Figure 1 and Figure 7.

Large Peritoneal Macrophages			
**Lineage Marker**	**WT**	**$12/15\text{-}LOX^{-/-}$**	**$12/15\text{-}LOX^{-/-}$ + $PD1_{n\text{-}3\ DPA}$**
TIM 4	11479.5 ± 1285.4	7487.1 ± 1108.7 *	10738.4 ± 1767.6
CD64	5112.7 ± 460.6	5299.6 ± 478.1	5267.6 ± 802.9
TGFβ	924.9 ± 112.0	647.1 ± 124.2	1106.9 ± 162.8 #
MHCII	10338.4 ± 772.9	9914.0 ± 1200.7	9471.4 ± 1791.3
Arg-1	1192.0 ± 254.3	2004.1 ± 223.5 *	1398.8 ± 333.3
IL-10	621.4 ± 86.9	911.1 ± 46.0 **	787.4 ± 113.8
CD11b	7844.2 ± 856.0	11380.1 ± 737.9 **	9023.9 ± 1028.7 #

Small Peritoneal Macrophages			
**Lineage Marker**	**WT**	**$ALOX15^{-/-}$**	**$ALOX15^{-/-}$ + $PD1_{n\text{-}3\ DPA}$**
TIM-4	1741.6 ± 160.8	1129.3 ± 175.9 *	1680.4 ± 313.8
CD64	7908.3 ± 569.2	10092.6 ± 698.8 *	8196.0 ± 984.2
TGFβ	117.2 ± 7.6	181.5 ± 15.4 **	140.1 ± 12.7 #
MHCII	5972.0 ± 250.4	4923.7 ± 335.9 *	5943.4 ± 767.6
Arg-1	4284.9 ± 354.0	3356.3 ± 219.6 *	4160.4 ± 544.7
IL-10	365.1 ± 25.7	452.6 ± 41.4	357.9 ± 14.2 #
CD11b	296952.6 ± 111533.7	125432.1 ± 30807.4	490012.7 ± 204253.1 #

Splenic Macrophages			
**Lineage Marker**	**WT**	**$ALOX15^{-/-}$**	**$ALOX15^{-/-}$ + $PD1_{n\text{-}3\ DPA}$**
PTGS2	701.0 ± 22.2	894.7 ± 4.4 **	792.3 ± 14.4 ##
iNOS	3822.8 ± 273.1	4589.0 ± 184.4	4066.7 ± 290.0
CD64	7528.5 ± 468.8	9644.3 ± 292.0 *	8484.7 ± 315.6
TGFβ	72.2 ± 9.3	118.7 ± 7.2 *	96.2 ± 9.9
MHCII	11416.3 ± 461.6	10378.7 ± 369.3	10847.0 ± 247.2
CD11c	347.8 ± 6.0	501.0 ± 33.6 *	405.7 ± 18.8
Arg-1	70.6 ± 7.6	170.0 ± 10.6 **	129.3 ± 4.1 #
IL-10	123.0 ± 2.0	154.7 ± 2.2 **	136.7 ± 1.7 #
CD11b	2129.0 ± 172.6	4029.7 ± 174.9 **	2476.7 ± 92.3 #

Tissue resident macrophages were collected from WT mice given vehicle (PBS) or $ALOX^{-/-}$ mice given either vehicle of 10ng $PD1_{n\text{-}3\ DPA}$ daily for 7 days. The expression of lineage markers was determined using flow cytometry and fluorescently labeled antibodies. Results are mean ± SEM n=7 mice per group for Large and small peritoneal macrophages n= 7 and splenic macrophages n=4 mice per group. * P < 0.05; ** P <0.01 vs WT vehicle group; # P < 0.05; ** P <0.01 vs ALOX15 vehicle group.
