# Supplementary material for: PDn-3 DPA Pathway Regulates Human Monocyte Differentiation and Macrophage Function
Source: Cell Chem Biol. 2018 Jun 21;25(6):749–760.e9. doi: 10.1016/j.chembiol.2018.04.017 (PMC6024030; doi:10.1016/j.chembiol.2018.04.017)
Supplement: Document S2. Article plus Supplemental Information [file mmc2.pdf]

Article

Cell Chemical Biology

$PD_{n\text{-}3\ DPA}$ Pathway Regulates Human Monocyte Differentiation and Macrophage Function

Graphical Abstract

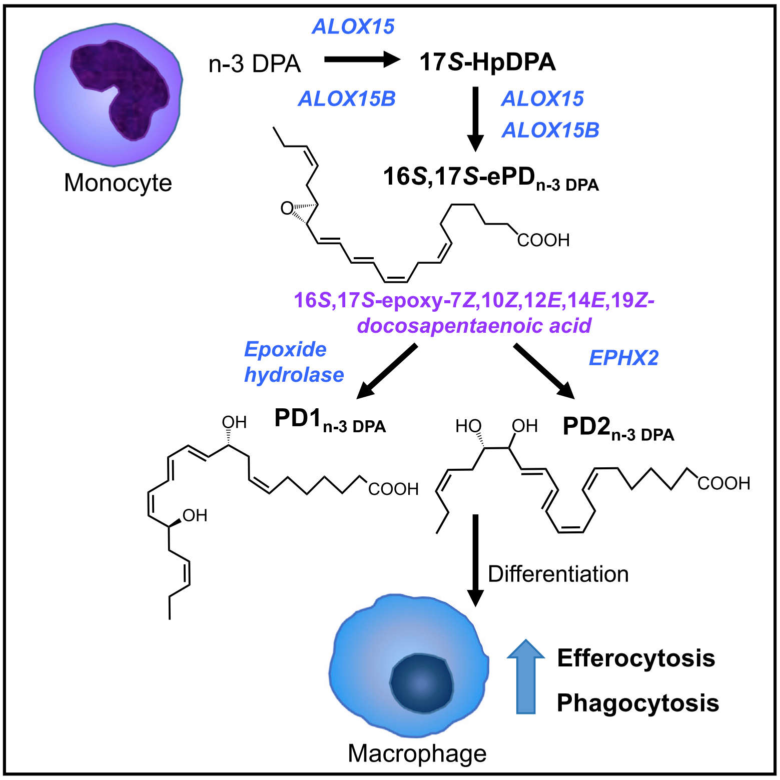

Authors

Kimberly Pistorius, Patricia R. Souza, Roberta De Matteis, ..., Raquel M. Marques, Trond V. Hansen, Jesmond Dalli

Correspondence

j.dalli@qmul.ac.uk

In Brief

Pistorius et al., found that a recently uncovered family of bioactive mediators termed $PD_{n\text{-}3\ DPA}$ controls human macrophage phenotype and function during their differentiation from monocytes. The authors also established the biosynthetic pathway for this family of mediators thereby providing leads into mechanisms that control macrophage responses.

Highlights

- $PD_{n\text{-}3\ DPA}$ regulates human monocyte-derived macrophage differentiation and function
- Evidence for the formation and complete stereochemistry of 16*S*,17*S*-$ePD_{n\text{-}3\ DPA}$
- EPHX2 converts 16*S*,17*S*-$ePD_{n\text{-}3\ DPA}$ to $PD2_{n\text{-}3\ DPA}$ in human monocytes

Pistorius et al., 2018, Cell Chemical Biology *25*, 749–760
June 21, 2018 © 2018 The Authors. Published by Elsevier Ltd.
https://doi.org/10.1016/j.chembiol.2018.04.017

CellPress

Cell Chemical Biology
Article

CellPress

$PD_{n-3\ DPA}$ Pathway Regulates Human Monocyte Differentiation and Macrophage Function

Kimberly Pistorius,[1] Patricia R. Souza,[1] Roberta De Matteis,[1] Shani Austin-Williams,[1] Karoline G. Primdahl,[2] Anders Vik,[2] Francesca Mazzacuva,[1] Romain A. Colas,[1] Raquel M. Marques,[1] Trond V. Hansen,[2] and Jesmond Dalli[1,3,*]
[1]William Harvey Research Institute and John Vane Science Centre, Barts and The London School of Medicine and Dentistry, Queen Mary University of London, Charterhouse Square, London EC1M 6BQ, UK
[2]School of Pharmacy, Department of Pharmaceutical Chemistry, University of Oslo, P.O. Box 1068 Blindern, Oslo 0316, Norway
[3]Lead Contact
*Correspondence: j.dalli@qmul.ac.uk
https://doi.org/10.1016/j.chembiol.2018.04.017

SUMMARY

Macrophages are central in orchestrating the clearance of apoptotic cells and cellular debris during inflammation, with the mechanism(s) regulating this process remaining of interest. Herein, we found that the n-3 docosapentaenoic acid-derived protectin ($PD_{n-3\ DPA}$) biosynthetic pathway regulated the differentiation of human monocytes, altering macrophage phenotype, efferocytosis, and bacterial phagocytosis. Using lipid mediator profiling, human primary cells and recombinant enzymes we found that human 15-lipoxygenases initiate the $PD_{n-3\ DPA}$ pathway catalyzing the formation of an allylic epoxide. The complete stereochemistry of this epoxide was determined using stereocontrolled total organic synthesis as 16*S*,17*S*-epoxy-7*Z*,10*Z*,12*E*,14*E*,19*Z*-docosapentaenoic acid (16*S*,17*S*-e$PD_{n-3\ DPA}$). This intermediate was enzymatically converted by epoxide hydrolases to $PD1_{n-3\ DPA}$ and $PD2_{n-3\ DPA}$, with epoxide hydrolase 2 converting 16*S*,17*S*-e$PD_{n-3\ DPA}$ to $PD2_{n-3\ DPA}$ in human monocytes. Taken together these results establish the $PD_{n-3\ DPA}$ biosynthetic pathway in human monocytes and macrophages and its role in regulating macrophage resolution responses.

INTRODUCTION

Macrophages are central in orchestrating host responses to tissue injury and infections (Fredman et al., 2014; Gordon and Mantovani, 2011; Pierdomenico et al., 2015). The biological actions of these cells are associated with their phenotype, where classically activated macrophages are central in the initiation and propagation of the inflammatory response, whereas alternatively activated macrophages orchestrate tissue repair and regeneration (Dalli and Serhan, 2016; Gordon and Mantovani, 2011; Morris et al., 2010). Investigations into the cellular mechanisms underlying these distinct biological functions demonstrate that each macrophage subtype produces a characteristic repertoire of bioactive molecules, including cytokines and lipid mediators (LMs) (Dalli and Serhan, 2016; Gordon and Mantovani, 2011). In this context, recent studies demonstrate that, while classically activated macrophages and alternatively activated macrophages produce both inflammation initiating eicosanoids and pro-resolving mediators, their LM phenotypes differ. Indeed, classically activated macrophages produce higher amounts of pro-inflammatory eicosanoids, whereas alternatively activated macrophages produce higher levels of the recently identified specialized pro-resolving mediators (SPMs) (Dalli and Serhan, 2012). Tissue macrophages arise during either embryonic development or are differentiated *in situ* from monocyte precursors (Kopf et al., 2015). Mechanisms that dictate monocyte differentiation to different macrophage phenotypes are of relevance in determining whether inflammation is perpetuated or resolves, thus permitting regain of function.

SPMs are produced via the stereoselective conversion of essential fatty acids. The first SPMs identified were the lipoxins (LX) that are produced from arachidonic acid and carry potent leukocyte directed actions (Serhan et al., 1984). The D-series resolvins (RvD) produced from docosahexaenoic acid (DHA) and the E-series resolvins (RvE) produced from eicosapentaenoic acid also display leukocyte-directed actions (Chiang and Serhan, 2017). In addition, RvEs regulate platelet response (Dona et al., 2008) and neutrophil apoptosis (El Kebir et al., 2012), while the RvDs regulate vascular smooth muscle cell migration, a key process in the development of intimal hyperplasia (Mottola et al., 2017). Recently, we found that n-3 docosapentaenoic acid (DPA) is also a substrate for conversion to a new series of pro-resolving mediators, which include the n-3 DPA-derived protectins ($PD_{n-3\ DPA}$) (Dalli et al., 2013a). These mediators were initially identified in self-limited inflammatory exudates, with their production coinciding with monocyte/macrophage trafficking to the site. Human primary macrophages also produce these mediators. Of note, $PD1_{n-3\ DPA}$ and $PD2_{n-3\ DPA}$ carry potent leukocyte directed actions, reducing neutrophil recruitment as well as the production of inflammatory cytokines, including monocyte chemoattractant protein-1, during sterile inflammation (Dalli et al., 2013a). These actions were also found with human primary cells, where $PD_{n-3\ DPA}$ reduce human neutrophil-endothelial cell interactions, neutrophil chemotaxis, and increased macrophage phagocytosis. The complete stereochemistry of the first member of this family of mediators, $PD1_{n-3\ DPA}$, was recently established as 10*R*,17*S*-dihydroxydocosa-7*Z*,11*E*,13*E*,15*Z*,19*Z*-pentaenoic acid (Aursnes et al., 2014). In humans, $PD1_{n-3\ DPA}$ is upregulated in colon samples

This is an open access article under the CC BY license (http://creativecommons.org/licenses/by/4.0/).

CellPress

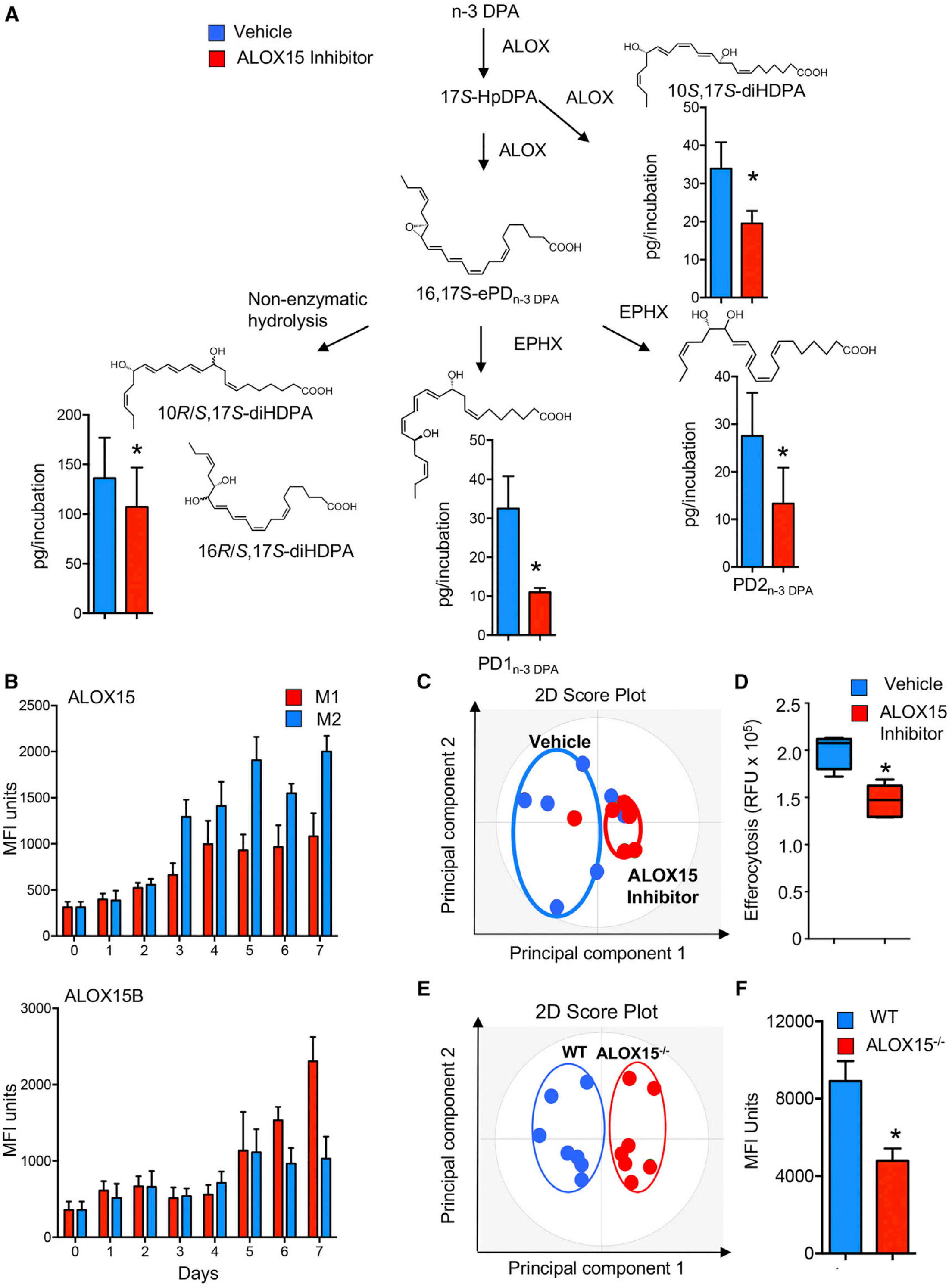

Figure 1. Inhibiting 15-Lipoxygenase Activity Reduces $PD_{n\text{-}3\ DPA}$ Production Dysregulating Macrophage Phenotype and Function

(A) Human monocytes were incubated with M-CSF (20 ng/mL) and either a ALOX15 inhibitor or vehicle (37°C, 5% CO_2). On day 7 incubations were quenched, lipid mediators were extracted, identified, and quantified using lipid mediator profiling (see the STAR Methods for details). Results are mean ± SEM. n = 6 donors. *$p < 0.05$.

(B) Human monocytes were isolated and incubated with GM-CSF (20 ng/mL), IFN-γ (20 ng/mL), and LPS (100 ng/mL) to produce M1 or M-CSF (20 ng/mL) and IL-4 (20 ng/mL) to obtain M2 cells, and the expression of ALOX15 and ALOX15B was evaluated during the differentiation time course using flow cytometry. Results are mean ± SEM n = 4–6 donors per interval.

(legend continued on next page)

from IBD patients and displays potent tissue protective actions in mice during experimental colitis, reducing leukocyte-mediated tissue damage (Gobbetti et al., 2017).

The $PD_{n-3\ DPA}$ biosynthetic pathway in human monocytes and macrophages is proposed to be initiated by 15-lipoxygenases (LOX), which convert n-3 DPA to an intermediate epoxide that in turn is enzymatically hydrolyzed to $PD1_{n-3\ DPA}$ (Aursnes et al., 2014) and $PD2_{n-3\ DPA}$ (16,17*R*-dihydroxy-7*Z*,10,13,14,19*Z*-docosapentaenoic acid) (Dalli et al., 2013a). Given the potent biological actions of $PD_{n-3\ DPA}$, and the central role of the proposed epoxide intermediate in the formation of these pro-resolving mediators, it was deemed important to (1) obtain evidence for its formation and role in the $PD_{n-3\ DPA}$ biosynthesis, (2) establish the identity of enzyme(s) involved in the formation of this epoxide in human monocytes/macrophages, and (3) establish the biological actions of the $PD_{n-3\ DPA}$ pathway in monocyte differentiation and macrophage responses. Using acid alcohol trapping, recombinant enzymes and human primary cells we found that both ALOX15 and ALOX15B catalyze the formation of 16,17*S*-$ePD_{n-3\ DPA}$. Using total organic synthesis we obtained stereochemically pure material, that, when incubated with primary human macrophages, was converted to both $PD1_{n-3\ DPA}$ and $PD2_{n-3\ DPA}$. Furthermore, we found that the $PD_{n-3\ DPA}$ pathway was important in regulating macrophage phenotype and function.

RESULTS

Inhibition of 15-Lipoxygenase Activity in Human Monocytes Reduced Macrophage $PD_{n-3\ DPA}$ Metabolome and Altered Macrophage Function

To obtain evidence for the role of the $PD_{n-3\ DPA}$ biosynthetic pathway in regulating human macrophage function during monocyte differentiation, we incubated monocytes with an ALOX15 inhibitor and assessed the production of components within the $PD_{n-3\ DPA}$ pathway. In human primary cells, using liquid chromatography-tandem mass spectrometry (LC/MS-MS), we identified 17-HDPA, $PD1_{n-3\ DPA}$, and $PD2_{\ n-3\ DPA}$. In these incubations we also identified 10*R*/*S*,17*S*-diHDPA and 16*R*/*S*,17*S*-diHDPA, which correspond to the non-enzymatic products of the proposed epoxide intermediate in the $PD_{n-3\ DPA}$ pathway (Figure 1A). Of note, incubation of monocytes with an inhibitor of ALOX15 led to a significant reduction in all the components of the $PD_{n-3\ DPA}$ biosynthetic pathway, including the bioactive mediators $PD1_{n-3\ DPA}$ and $PD2_{n-3\ DPA}$ (Figure 1A; Table S1). The role of 15-lipoxygenases in $PD_{n-3\ DPA}$ biosynthesis was further corroborated using cells from mice deficient in the murine homolog of this enzyme ($ALOX15^{-/-}$), which demonstrated a significant reduction in all the components within the $PD_{n-3\ DPA}$ pathway (Figure S1; Table S2).

Human leukocytes express two ALOX15 subtypes, therefore we sought to determine the temporal regulation of these enzymes in circulating monocytes and during monocyte-macrophage differentiation. Flow cytometric analysis demonstrated that human circulating monocytes express both ALOX15 and ALOX15B. The expression of both enzyme isoforms was upregulated during monocyte-to-macrophage differentiation. Of note, ALOX15 expression was higher in M2 differentiated cells, whereas the expression of ALOX15B was higher in M1 cells (Figure 1B). We next investigated the contribution of each of these two enzymes to $PD_{n-3\ DPA}$ biosynthesis in human monocytes. Here we found that transfection of human monocytes with short hairpin RNA (shRNA) to ALOX15 reduced enzyme expression by ~50% ($p < 0.05$; n = 5 donors), and the concentrations of several components of the $PD_{n-3\ DPA}$ biosynthetic pathway, including $PD1_{n-3\ DPA}$ and $PD2_{n-3\ DPA}$. Transfection of monocytes with shRNA to ALOX15B significantly reduced both enzyme expression (~50%; $p < 0.05$; n = 5 donors) and $PD_{n-3\ DPA}$ concentrations, albeit to a lesser extent than observed with shRNA to ALOX15 (Figure S2).

Given that $PD1_{n-3\ DPA}$ and $PD2_{n-3\ DPA}$ carry potent biological actions, we next questioned whether inhibition of this pathway during monocyte-to-macrophage differentiation influenced macrophage phenotype and function. We therefore assessed the expression of phagocytic receptors and adhesion molecules, which are linked with macrophage phenotype. Incubation of cells with ALOX15 inhibitor during monocyte differentiation led to the downregulation of several lineage markers, including CD206, CD163, and CD64, and a shift in macrophage phenotype (Figure 1C). This downregulation in phagocytic receptors was of functional consequence since inhibition of the $PD_{n-3\ DPA}$ biosynthetic pathway also significantly downregulated the ability of human macrophages to uptake apoptotic cells (Figure 1D), a key pro-resolving action (Chiang and Serhan, 2017; Dalli and Serhan, 2016). This alteration in macrophage phenotype and function was also observed in tissue-resident macrophages from ALOX15-deficient animals. In these mice the expression of lineage markers on splenic macrophages, small peritoneal macrophages, and large peritoneal macrophages was altered with a downregulation in the expression of several markers including transforming growth factor β and T-cell immunoglobulin and mucin domain containing protein (TIM)-4 (Figure 1E). This was associated with a decreased ability of peritoneal macrophages to uptake apoptotic cells *in vivo* (Figure 1F; Table S3).

Having established a role of the $PD_{n-3\ DPA}$ metabolome in regulating human macrophage function we next sought to obtain further evidence for the $PD_{n-3\ DPA}$ biosynthetic pathway. Given the central role that the proposed 16,17*S*-$ePD_{n-3\ DPA}$ plays in the biosynthesis of $PD_{n-3\ DPA}$, we sought to gain evidence for the formation of the proposed epoxide in human monocytes. For

(C and D) Human monocytes were incubated with vehicle or ALOX15 inhibitor and then with M-CSF (20 ng/mL) for 7 days, and (C) expression of lineage markers was determined using fluorescently labeled antibodies and flow cytometry on day 7, and interrogated using OPLS-DA. n = 6 donors. (D) Phagocytosis of fluorescently labeled apoptotic cells investigated. Results for are mean ± SEM. n = 6 donors. *$p < 0.05$.

(E) Peritoneal macrophages were harvested from wild-type (WT) and $ALOX15^{-/-}$ mice, and the expression of lineage markers on $CD64^{+}$ cells was determined using flow cytometry. Results were interrogated using OPLS-DA and are representative of n = 7 mice.

(F) Fluorescently labeled apoptotic cells were administered to WT and $ALOX15^{-/-}$ mice via intraperitoneal injection. After 1 hr peritoneal cells were harvested, and phagocytosis of apoptotic cells by $CD64^{+}$ cells was evaluated using flow cytometry. Results are mean ± SEM. n = 7 mice per group. *$p < 0.05$.

Related to Figures S1 and S2 and Tables S1–S3.

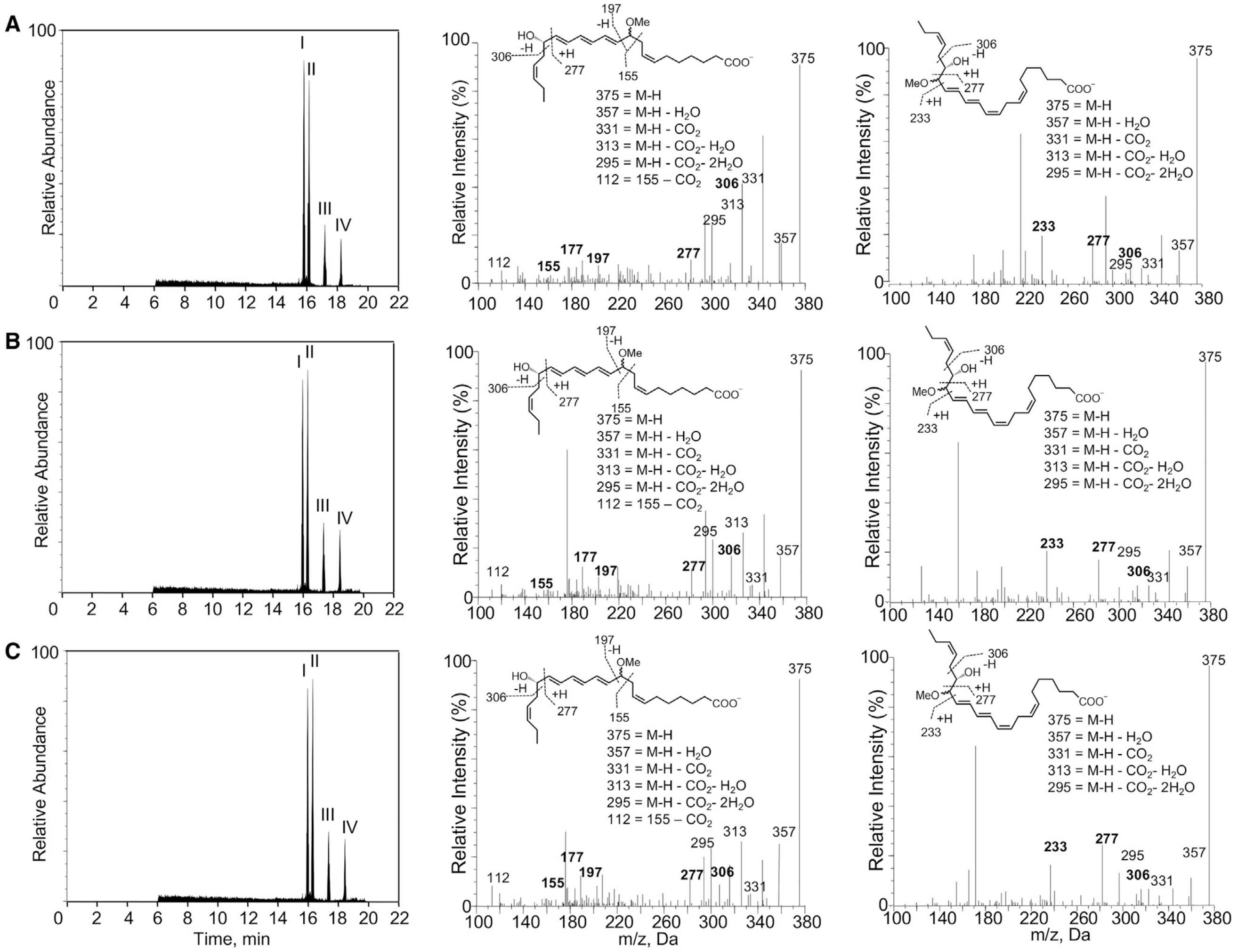

Figure 2. Human ALOX15 and Monocytes Produces a Novel 16,17*S*-ePD$_{n-3\ DPA}$

(A–C) Human monocytes (1×10^8 cells/mL; PBS; 37°C) (A), hr-ALOX15 (0.2 μM, 37°C [pH 8]) (B), and hr-ALOX15B (0.2 μM, 37°C [pH 8]) (C) were incubated with n-3 DPA (10 μM). After 3 min, incubations were quenched using acidified methanol, products extracted and identified using lipid mediator profiling. Left panels: MRM chromatogram for ion pairs *m/z* 375 > 277. Middle and right panels: MS-MS spectra employed in the identification of (middle panel) 10-methoxy,17*S*-hydroxy-7*Z*,11*E*,13*E*,15*E*,19Z-docosapentaenoic acid, (right panel) 16-methoxy,17*S*-hydroxy-7*Z*,10*Z*,12*E*,14*E*,19*Z*-docosapentaenoic acid in monocyte incubations. Results are representative of n = 4 donors and three independent experiments. Related to Figures S3 and S4.

this purpose we assessed the formation of the epoxide using acid alcohol trapping, given that allylic epoxides are known to be unstable in aqueous solutions (Radmark et al., 1980). Using LC/MS-MS we identified four peaks, the first two gave a retention time (T_R) and MS-MS fragmentation spectra corresponding with 10-methoxy,17*S*-hydroxy-docosapentaenoic acid (Figure 2A), whereas, the T_R and MS-MS spectra for peaks III and IV corresponded to 16-methoxy,17*S*-hydroxy-docosapentaenoic acid (Figure 2A).

Having obtained evidence for the formation of an epoxide intermediate by human monocytes we sought to determine whether ALOX15 enzymes were responsible for the formation of this intermediate. For this purpose we incubated human recombinant (hr) ALOX15 and hr-ALOX15B with n-3 DPA and assessed the acid alcohol trapping products using LC/MS-MS. This also gave four peaks that displayed essentially identical T_R and MS-MS fragmentation spectra to the products obtained with primary human macrophages (Figures 2B and 2C). Thus, these results establish the role of human ALOX15 enzymes in the formation of 16,17*S*-ePD$_{n-3\ DPA}$.

Establishing the Complete Stereochemistry of 16*S*,17*S*-ePD$_{n-3\ DPA}$

Having obtained evidence for the formation of 16,17*S*-ePD$_{n-3\ DPA}$ by human monocytes and the role of ALOX15 enzymes in its production, we next sought evidence for its role in the biosynthesis of PD1$_{n-3\ DPA}$ and PD2$_{n-3\ DPA}$. For this purpose we obtained stereochemically pure material using total organic synthesis. The methyl ester of 16*S*,17*S*-ePD$_{n-3\ DPA}$ was constructed via two key precursors (Figure 3A). These precursors were obtained using the following stereoselective reactions: a Katsuki-Sharpless epoxidation protocol (Kumar and Meshram, 2013) and one *Z*- and two *E*-selective Wittig reactions (Maryanoff and Reitz, 1989). The detailed description of the synthetic route

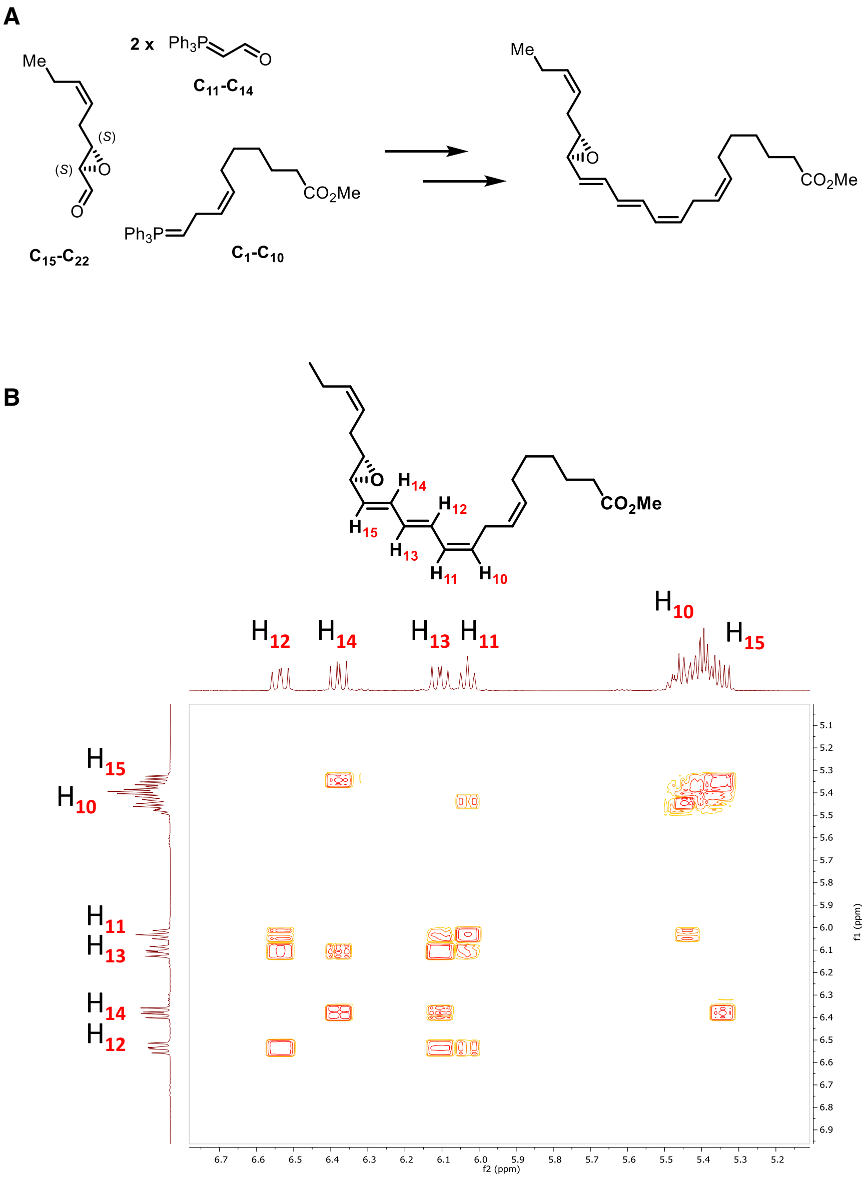

Figure 3. Total Organic Synthesis of 16,17*S*-ePD$_{n\text{-}3\ DPA}$

(A) Outline of the synthetic strategy and key precursors employed in the preparation of 16*S*,17*S*-ePD$_{n\text{-}3\ DPA}$.

(B) *Z* and *E* stereochemical assignment for C=C using two-dimensional NMR spectroscopy. Contours denote positive and negative contours. Related to Figures S3.

is reported in Primdahl et al. (2017). The chemical purity and stereochemical integrity was validated using LC/MS-MS and nuclear magnetic resonance (NMR). The unambiguous assignment of the geometrical configurations of the olefins constituting the *E,E,Z*-triene moiety was performed using a ^{1}H-^{1}H COSY-45 NMR experiment (Figures 3B and S3). The following chemical shifts and coupling constants (*J* values) were recorded for the epoxy methyl ester of 16*S*,17*S*-ePD$_{n\text{-}3\ DPA}$: H_{12}: 6.54 ppm, *J* = 14.7, 11.4 Hz; H_{14}: 6.39 ppm, *J* = 15.3, 10.8 Hz; H_{13}: 6.11 ppm, *J* = 14.8, 10.9 Hz; H_{11}: 6.04 ppm, *J* = 11.2 Hz; H_{10} and H_{15}: 5.48–5.32 ppm. Notably, H_{11} has a ^3J-coupling constant to H_{10} with a value of 11.2 Hz, which is consistent with the assigned *Z*-rather than an *E*-configured C10-C11 double bond. The UV chromophore of this compound was also consistent with that of an allylic epoxide conjugated to a triene double-bond system, giving a λ_{max}^{MeOH} of 280 nm with shoulders at 271 and 298 nm. These results confirm the stereochemistry of the synthetic epoxide as 16*S*,17*S*-epoxy-7*Z*,10*Z*,12*E*,14*E*,19*Z*-docosapentaenoic acid methyl ester.

We next assessed whether the physical properties of the synthetic material obtained after lenient hydrolysis of the epoxy methyl ester matched those of the biological material. Incubation of the synthetic material in acid alcohol gave four distinct peaks with products displaying essentially identical T_R and MS-MS fragmentation spectra as those obtained with hr-ALOX15 enzymes and human monocytes (Figures 2 and S4).

We next investigated whether the synthetic material was substrate for conversion to PD1$_{n\text{-}3\ DPA}$ and PD2$_{n\text{-}3\ DPA}$. To this end, human macrophages were incubated with 16*S*,17*S*-ePD$_{n\text{-}3\ DPA}$ and products were assessed using LC/MS-MS. In multiple reaction monitoring (MRM) we obtained two peaks. The first peak eluted with a T_R of 13.8 min and gave characteristic fragments in the MS-MS that are consistent with PD1$_{n\text{-}3\ DPA}$. These fragments included the following diagnostic ions: *m/z* 361, *m/z* 183, and *m/z* 155 (Figures 4A and 4B). The second peak eluted with a T_R of 15.2 min and gave ions in the MS-MS that were characteristic of PD2$_{n\text{-}3\ DPA}$ including: *m/z* 361, *m/z* 263, and *m/z* 233 (Figures 4A and 4C). Of note, incubation of 16*S*,17*S*-ePD$_{n\text{-}3\ DPA}$ in PBS only or with cells that had been previously kept at 100°C did not yield notable levels of these mediators (Figure 4D), indicating that the conversion of the epoxide to PD1$_{n\text{-}3\ DPA}$ and PD2$_{n\text{-}3\ DPA}$ was reliant on enzyme-mediated catalysis. In addition, these results establish the stereochemistry of the allylic epoxide intermediate as 16*S*,17*S*-epoxy-7*Z*,10*Z*,12*E*,14*E*,19*Z*-docosapentaenoic acid.

Epoxide Hydrolases Convert 16*S*,17*S*-ePD$_{n\text{-}3\ DPA}$ to PD1$_{n\text{-}3\ DPA}$ and PD2$_{n\text{-}3\ DPA}$

Having established a role for enzymes in catalyzing the conversion of 16*S*,17*S*-ePD$_{n\text{-}3\ DPA}$ to PD1$_{n\text{-}3\ DPA}$ and PD2$_{n\text{-}3\ DPA}$ we next sought to establish the identity of these enzymes. Given the role that epoxide hydrolases play in converting allylic epoxides to bioactive mediators we assessed whether

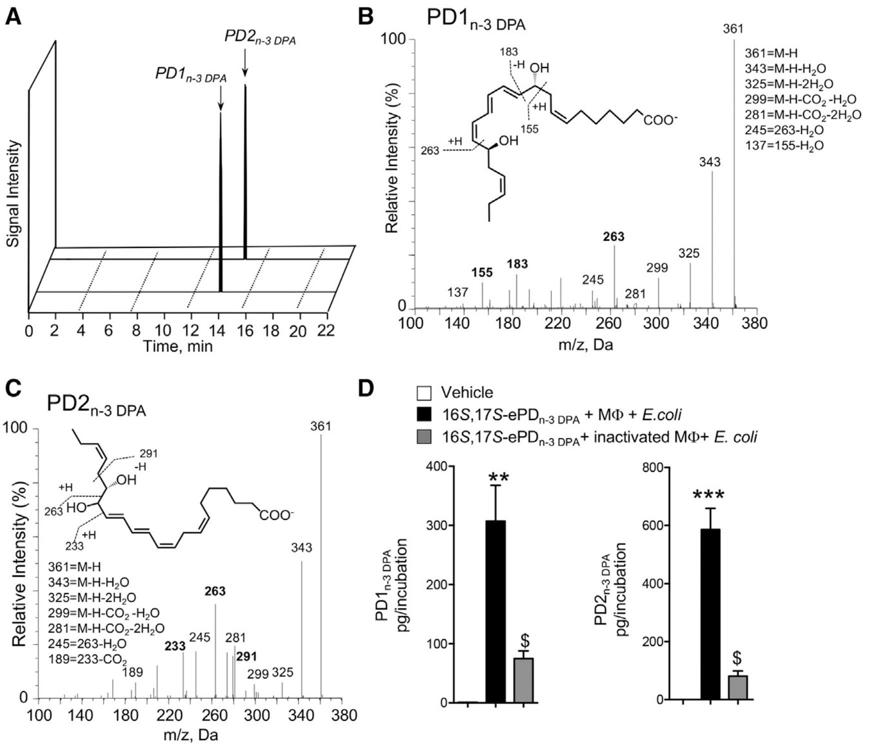

Figure 4. 16*S*,17*S*-ePD$_{n\text{-}3\ DPA}$ Is Precursor to PD1$_{n\text{-}3\ DPA}$ and PD2$_{n\text{-}3\ DPA}$

16*S*,17*S*-ePD$_{n\text{-}3\ DPA}$ (10 nM) was incubated with human macrophages (MΦ; 4 × 10^7 cells/mL) or inactivated human macrophages (i.e., 4 × 10^7 cells/mL previously been kept at 100°C for 1h). *E. coli* (2.5 × 10^8 colony-forming units [CFU]/mL) were added, cells incubated for 15 min, at 37°C, and incubations were quenched using ice-cold methanol. Products were then extracted and profiled using lipid mediator profiling. Vehicle denotes solution containing 0.1% EtOH in PBS.

(A) MRM chromatogram for PD1$_{n\text{-}3\ DPA}$ (*m/z* 361 > 183) and PD2$_{n\text{-}3\ DPA}$ (*m/z* 361 > 233).

(B and C) MS-MS spectra employed for identification of (B) PD1$_{n\text{-}3\ DPA}$ (C) PD2$_{n\text{-}3\ DPA}$.

(D) PD1$_{n\text{-}3\ DPA}$ and PD2$_{n\text{-}3\ DPA}$ concentrations. Results are representative of n = 4 donors from two independent experiments. Results are means ± SEM. **$p < 0.001$, ***$p < 0.0001$ versus vehicle incubations. \$p < 0.05 versus MΦ + *E. coli* incubations.

this class of enzymes was also involved in catalyzing the conversion of 16*S*,17*S*-ePD$_{n\text{-}3\ DPA}$ in human monocytes. Incubation of human macrophages with the epoxide hydrolase inhibitor AUDA led to a reduction in the concentrations of both PD1$_{n\text{-}3\ DPA}$ and PD2$_{n\text{-}3\ DPA}$ (Figure 5A).

Leukotriene A_4 hydrolase (LTA$_4$H) catalyses the hydrolysis of the allylic epoxide in LTA$_4$ at the least sterically hindered carbon of the carbocation intermediate (Radmark et al., 1980; Rudberg et al., 2002) and is also expressed by human macrophages (Qiu et al., 2006). Therefore we questioned whether this enzyme was also responsible for catalyzing the conversion of 16*S*,17*S*-ePD$_{n\text{-}3\ DPA}$ to PD1$_{n\text{-}3\ DPA}$. LC/MS-MS analysis of products obtained when LTA$_4$H was incubated with

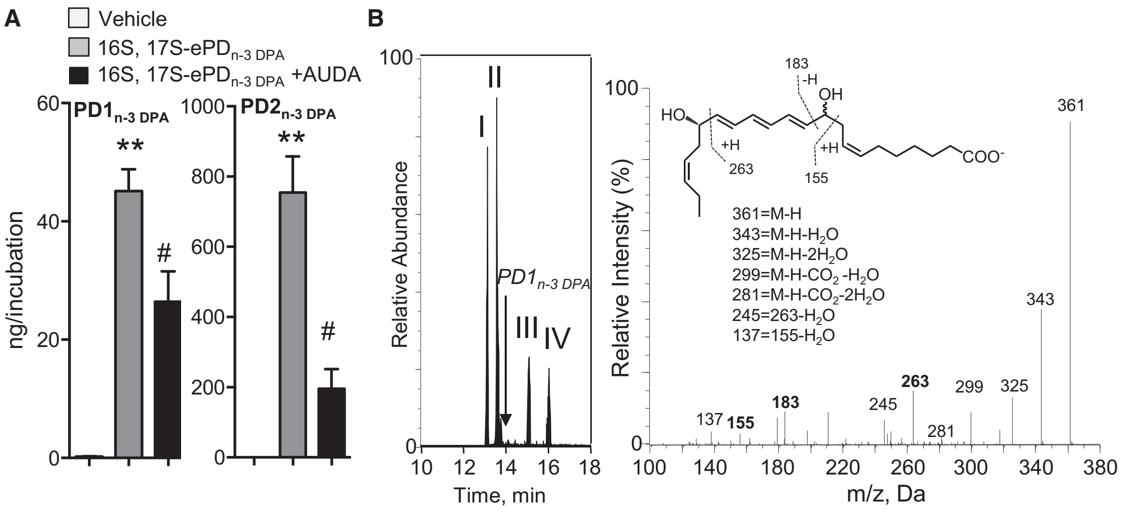

Figure 5. Epoxide Hydrolases Convert 16*S*,17*S*-ePD$_{n\text{-}3\ DPA}$ to PD1$_{n\text{-}3\ DPA}$ and PD2$_{n\text{-}3\ DPA}$

(A) Human monocytes (1 × 10^8 cells/mL) were incubated with vehicle (PBS + 0.1% DMSO) or AUDA (25 μM) for 20 min (at room temperature). Cells were then incubated with either vehicle (PBS + 0.1% EtOH) or 16*S*,17*S*-ePD$_{n\text{-}3\ DPA}$ (10 nM). Incubations were quenched after 15 min and products profiled using LM profiling. Results are mean ± SEM. n = 4 donors and two independent experiments. **$p < 0.01$ versus vehicle; #$p < 0.05$ versus monocyte incubations.

(B) 16*S*,17*S*-ePD$_{n\text{-}3\ DPA}$ (10 nM) was incubated with human recombinant LTA$_4$H (0.2 μM; Tris buffer). Incubations were quenched using ice-cold methanol and products identified using lipid mediator profiling. Left panel: MRM chromatogram *m/z* 361 > 263 (arrow denotes expected retention time for PD1$_{n\text{-}3\ DPA}$); right panel: MS-MS spectrum employed in the identification of 10,17*S*-hydroxy-7*Z*,11*E*,13*E*,15*E*,19*Z*-docosapentanenoic acid. Results are representative of n = 4 independent experiments.

CellPress

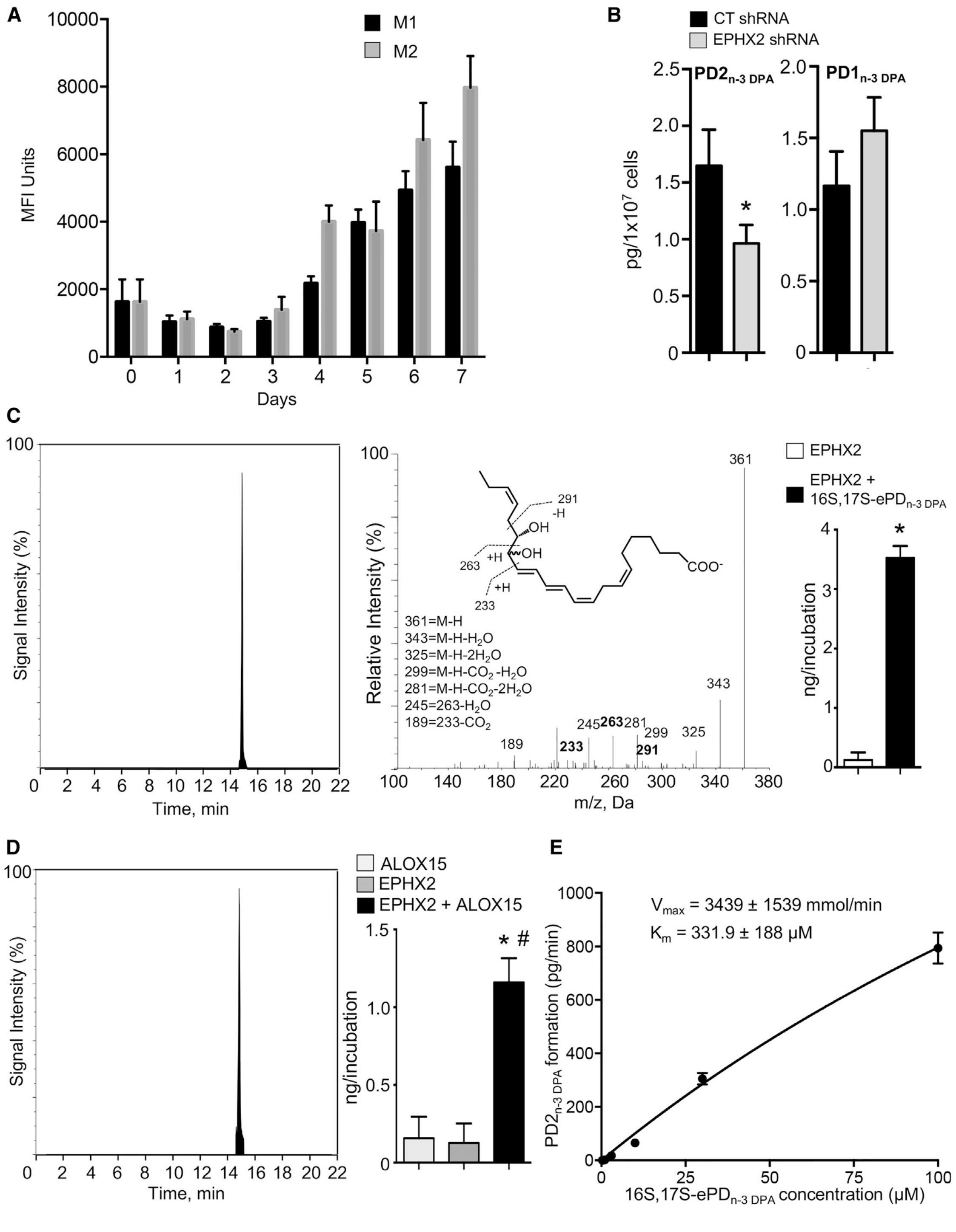

Figure 6. EPHX2 Converts 16*S*,17*S*-ePD$_{n\text{-}3\ DPA}$ to PD2$_{n\text{-}3\ DPA}$ in Human Monocytes and Macrophages

(A) Human monocytes were isolated and differentiated using GM-CSF (20 ng/mL), IFN-γ (20 ng/mL), and LPS (100 ng/mL) to produce M1 or M-CSF (20 ng/mL) and IL-4 (20 ng/mL) to obtain M2 cells and the expression of EPHX2 during the differentiation time course was evaluated using flow cytometry. Results are mean ± SEM. n = 4–6 donors per interval.

(B) Human monocytes were transfected with shRNA to EPHX2 or CT shRNA (see the STAR Methods for details), cells were incubated for 10 hr at 37°C, then with *E. coli* for 45 min, and PD$_{n\text{-}3\ DPA}$ concentrations evaluated using LM profiling. Results are mean ± SEM. n = 4 donors. $^*p < 0.05$.

(C) 16*S*,17*S*-ePD$_{n\text{-}3\ DPA}$ (10 nM) was incubated with hrEPHX2 (0.2 μM; Tris buffer). Incubations were quenched using ice-cold methanol and products identified using lipid mediator profiling. Left panel*:* MRM chromatogram *m/z* 361 > 233. Center panel*:* MS-MS spectrum employed in the identification of PD2$_{n\text{-}3\ DPA}$. Right panel: PD2$_{n\text{-}3\ DPA}$ concentrations. Results are representative of n = 4 independent experiments. $^*p < 0.01$ versus EPHX2 incubations.

(legend continued on next page)

16*S*,17*S*-ePD$_{n-3\ DPA}$ gave four peaks that were identified as the non-enzymatic hydrolysis products of 16*S*,17*S*-epoxy-PD$_{n-3\ DPA}$. The T_R and MS-MS spectra of peaks I and II corresponded to 10*R*/*S*,17*S*-dihydroxy-7*Z*,11*E*,13*E*,15*E*,19*Z*-docosapentaenoic acid, whereas peaks III and IV were identified as 16*R*/*S*,17*S*-dihydroxy-7*Z*,10*Z*,12*E*,14*E*,19*Z*-docosapentaenoic acid (Figure 5B). For comparison LM profiling of the products obtained when LTA_4H was incubated with LTA_4 gave a major peak that eluted with a T_R of 13.6 min, with the MS-MS spectrum corresponding to LTB_4 (n = 4 incubations).

Another epoxide hydrolase expressed by human macrophages, which is also involved in the catalysis of allylic epoxides to bioactive mediators, is epoxide hydrolase 2 (EPHX2) (Deng et al., 2014). Thus, we next investigated whether this enzyme may play a role in the PD$_{n-3\ DPA}$ biosynthetic pathway. Using flow cytometry we found that EPHX2 is expressed in monocytes and is upregulated during monocyte-to-macrophage differentiation (Figure 6A). Transfection of human monocytes with shRNA to EPHX2 led to a significant downregulation of PD2$_{n-3\ DPA}$ and an increase in PD1$_{n-3\ DPA}$ concentrations (Figure 6B). To further validate the role of EPHX2 in PD2$_{n-3\ DPA}$ biosynthesis, we incubated hr-EPHX2 with 16*S*,17*S*-ePD$_{n-3\ DPA}$, which gave a peak with T_R of 15.2 min and an MS-MS spectrum that corresponded to that of PD2$_{n-3\ DPA}$ (Figure 6C). Of note, in these incubations we did not identify PD1$_{n-3\ DPA}$, suggesting that this enzyme selectively catalyzed the conversion of 16*S*,17*S*-ePD$_{n-3\ DPA}$ to PD2$_{n-3\ DPA}$. Having found that hr-EPHX2 converted the epoxide to PD2$_{n-3\ DPA}$, we next co-incubated hr-ALOX15 with hr-EPHX2 to obtain further evidence for the PD2$_{n-3\ DPA}$ biosynthetic pathway in human cells. LM profiling of these incubations gave a peak with T_R and MS-MS spectrum corresponding to PD2$_{n-3\ DPA}$ (Figure 6D). Of note, levels of this mediator in incubations with either hr-ALOX15 or EPHX2 were negligible. Similar findings were also made with incubations of hr-ALOX15B and EPHX2 (n = 3 distinct incubations). To further assess the role of EPHX2 in the biosynthesis of PD2$_{n-3\ DPA}$ we incubated this enzyme with increasing concentrations of 16*S*,17*S*-ePD$_{n-3\ DPA}$ and determined the reaction kinetics. Here we found that 16*S*,17*S*-ePD$_{n-3\ DPA}$ was rapidly converted with a V_{max} of 3,439 ± 1,539 mmol/min and a K_M of 332 ± 188 μM (Figure 6E). Together these findings demonstrate that epoxide hydrolase enzymes are key in the conversion of 16*S*,17*S*-ePD$_{n-3\ DPA}$ to PD1$_{n-3\ DPA}$ and PD2$_{n-3\ DPA}$, and identify EPHX2 as the enzyme responsible for the conversion of 16*S*,17*S*-ePD$_{n-3\ DPA}$ to PD2$_{n-3\ DPA}$.

PD$_{n-3\ DPA}$ Regulate Human Macrophage Phenotype and Responses

Having found that inhibition of the PD$_{n-3\ DPA}$ pathway alters macrophage phenotype we next sought to determine the role of select members of the PD$_{n-3\ DPA}$ pathway during monocyte-to-macrophage differentiation. Co-incubation of human macrophages with an ALOX15 inhibitor and either 16*S*,17*S*-ePD$_{n-3\ DPA}$ or PD1$_{n-3\ DPA}$ upregulated the expression of several lineage markers, including CD163 and CD64, when compared with cells incubated with the inhibitor alone (Figure S5A). We next investigated whether the upregulation of these lineage markers was also associated with a restoration of the ability of human macrophages to clear apoptotic cells. Indeed, incubation of monocyte-dervied macrophages with 16*S*,17*S*-ePD$_{n-3\ DPA}$ or PD1$_{n-3\ DPA}$ rectified their ability to uptake apoptotic cells (Figures S5B and S5C). These restorative actions of PD$_{n-3\ DPA}$ on macrophage function were also retained *in vivo*, where administration of PD1$_{n-3\ DPA}$ rectified the expression of lineage markers on both splenic and peritoneal macrophages from ALOX15$^{-/-}$ mice (Figures 7A–7C; Table S3). Furthermore PD1$_{n-3\ DPA}$ also upregulated the ability of peritoneal macrophages from ALOX15$^{-/-}$ mice to uptake apoptotic cells (Figure 7D) and bacteria (Figure 7E) *in vivo*. Together, these results demonstrate that the PD$_{n-3\ DPA}$ pathway regulates key human macrophage functions in promoting the resolution of inflammation.

DISCUSSION

Herein, we established the PD$_{n-3\ DPA}$ biosynthetic pathway and the role of this pathway in regulating key macrophage responses during monocyte-to-macrophage differentiation. Results from the present experiments demonstrate that, in human and mouse monocytes and macrophages, 15-lipoxygenases are the initiating enzyme in the PD$_{n-3\ DPA}$ pathway catalyzing the formation of 17*S*-HpDPA and 16*S*,17*S*-ePD$_{n-3\ DPA}$. The epoxide is then converted *via* enzymatic hydrolysis to PD1$_{n-3\ DPA}$ and PD2$_{n-3\ DPA}$, whereby the conversion of 16*S*,17*S*-ePD$_{n-3\ DPA}$ to PD2$_{n-3\ DPA}$ is catalyzed by EPHX2. Inhibition of ALOX15 activity led to a significant alteration in the expression of lineage markers as well as the ability of macrophages to uptake apoptotic cells and bacteria, actions that were recovered by either 16*S*,17*S*-ePD$_{n-3\ DPA}$ or PD1$_{n-3\ DPA}$.

During acute inflammation, monocytes are recruited to the site where under ideal conditions, they differentiate to macrophages with a tissue protective phenotype; a process that is central in the resolution of inflammation as well as tissue repair and regeneration (Bystrom et al., 2008; Dalli and Serhan, 2016). Chronic, unresolved inflammation is associated with dysregulated monocyte differentiation leading to macrophages displaying a pro-inflammatory phenotype (Dalli and Serhan, 2016; Newson et al., 2014; Sica et al., 2000). The mechanisms dictating whether monocytes differentiate into a tissue reparative or pro-inflammatory phenotype remain of interest. In the present study, we found that inhibiting ALOX15 expression and activity using both genetic and pharmacological approaches in mice and human primary cells dysregulated both macrophage phenotype and function (Figure 1). Incubation of monocytes throughout their differentiation into macrophages

(D) n-3 DPA (10 μM; Tris buffer) was incubated with hr-ALOX15 (0.2 μM), EPHX2 (0.2 μM), or a combination of the two enzymes. The incubations were quenched after 15 min and products extracted, identified, and quantified using lipid mediator profiling. Left panel: MRM chromatogram *m/z* 361 > 233; right panel: PD2$_{n-3\ DPA}$ concentrations. Results are representative of n = 4 independent experiments. Results for right panels in (C) and (D) are means ± SEM. *p < 0.01 versus EPHX2 incubations; #p < 0.01 versus ALOX15 incubations.
(E) EPHX2 (0.2 μM, Tris buffer) was incubated with the indicated concentrations of 16S,17S-ePD$_{n-3\ DPA}$. Incubations were quenched and PD2$_{n-3\ DPA}$ concentrations were determined using lipid mediator profiling. Results are mean ± SEM. n = 3 independent experiments.

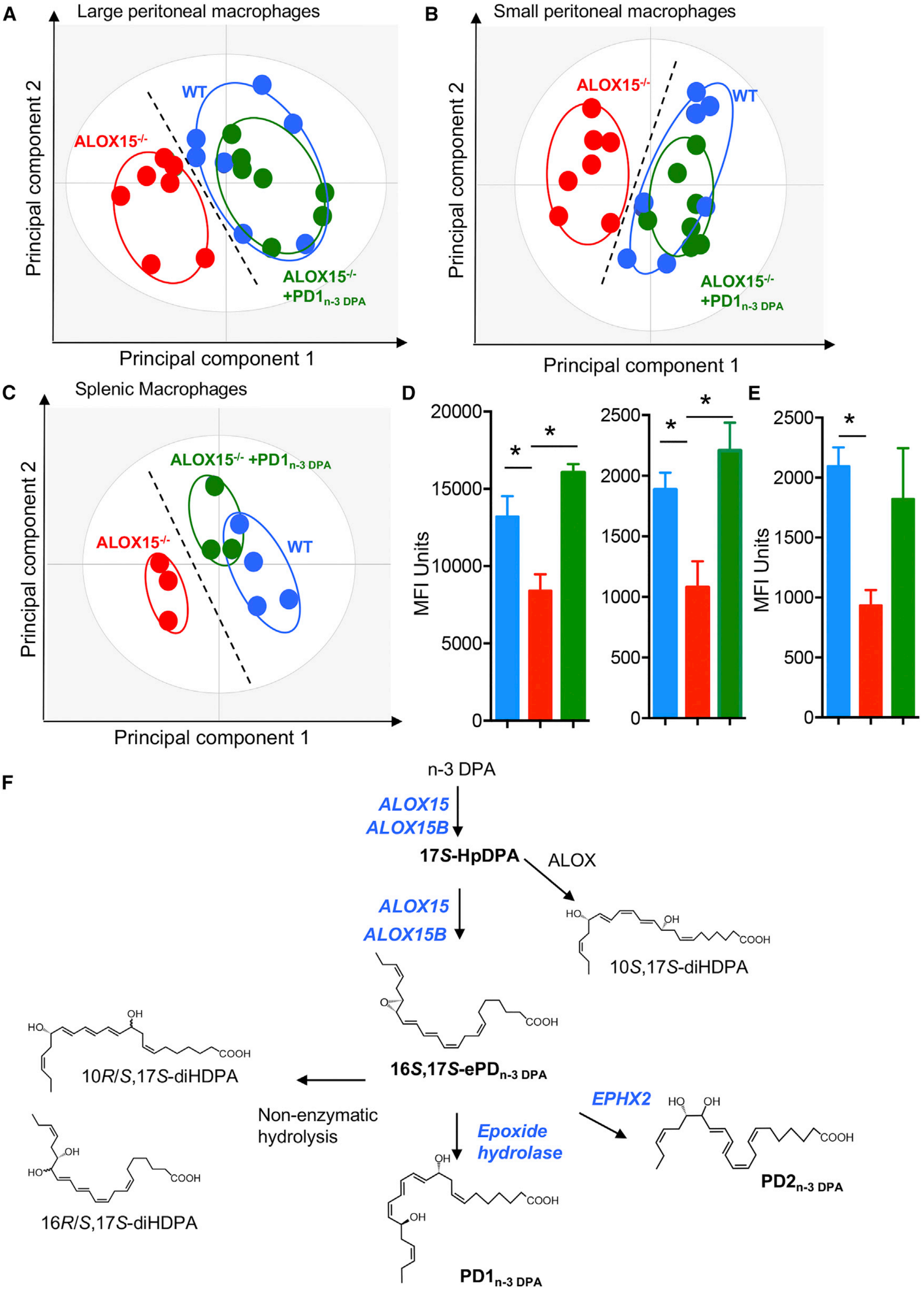

Figure 7. PD1$_{n\text{-}3\ DPA}$ Rectifies Murine Resident Macrophage Phenotype and Function in ALOX15-Deficient Mice

(A–C) The expression of phenotypic markers was assessed in peritoneal and splenic macrophages from ALOX15$^{-/-}$ mice administered PD1$_{n\text{-}3\ DPA}$ (10 ng/mouse for 7 days) or vehicle and WT mice using flow cytometry and macrophage phenotype interrogated using PLS-DA in (A) large peritoneal macrophages, (B) small peritoneal macrophages, and (C) splenic macrophages. Results are representative of n = 8 mice per group for (A and B) and n = 3–4 mice per group for (C).

(legend continued on next page)

with either 16*S*,17*S*-ePD$_{n\text{-}3\ DPA}$ or PD1$_{n\text{-}3\ DPA}$ rescued the ability of macrophages to uptake apoptotic cells as well as upregulated the expression of lineage markers, including CD64 and CD163, which were decreased following ALOX15 inhibition. PD1$_{n\text{-}3\ DPA}$ administration to ALOX15-deficient mice also rectified the expression of lineage markers on tissue-resident macrophages and the ability of peritoneal macrophages to uptake apoptotic cells and bacteria (Figure 7). Of note, ALOX15 is also the initiating enzyme in the RvD and LX biosynthetic pathway. Thus, while these results do not rule out the contribution of other ALOX15-derived pro-resolving mediators in the regulating monocyte-to-macrophage differentiation, they support a role for the PD$_{n\text{-}3\ DPA}$ metabolome in regulating this process. The present findings also suggest that 16*S*,17*S*-ePD$_{n\text{-}3\ DPA}$ is both a biosynthetic intermediate in the PD$_{n\text{-}3\ DPA}$ pathway and exerts independent biological actions regulating cellular functions. These results are in line with findings made with other allylic epoxides including LTA_4 (Ohishi et al., 1987) and 13*S*,14*S*-epoxy-maresin (Dalli et al., 2013c), which are also bioactive, regulating LM biosynthesis and cellular phenotype.

LMs are produced via the stereoselective conversion of essential fatty acids by their biosynthetic enzymes. Recent studies demonstrate that the differential regulation of the SPM biosynthetic enzymes may reflect disease status and contribute to the propagation of ongoing inflammation. In this context, for example, Fredman et al. (2014) found that phosphorylation of the ALOX5 changes the product profile of the enzyme, whereby phospho-ALOX5 translocates to the nuclear membrane where it couples with phospholipase A_2 and LTA_4H to produce the potent leukocyte chemoattractant LTB_4. In the absence of this phosphorylation the enzyme is found in the cytosol, where it couples with ALOX15, producing the tissue-protective and pro-resolving mediators RvD1 and LXA_4 (Fredman et al., 2014). These mechanisms are linked with disease onset/progression whereby, in atherosclerosis, recent studies identified an increased expression of phosphorylated ALOX5 and a decrease in the RvD1 to LTB_4 ratio in aortic lesions (Fredman et al., 2016). These results may also shed light on the apparently discordant results obtained with omega-3 supplementation. This is because, if the expression and/or subcellular localization of SPM biosynthetic enzymes is dysregulated, the product profile will be altered and the expected beneficial actions via SPM production may be diminished. Thus, furthering our understanding on the biosynthetic pathways governing SPM biosynthesis is essential in order to gain better insights into disease etiopathology. The present studies focus on establishing the role of the PD$_{n\text{-}3\ DPA}$ in cells of the monocytic lineage. These findings demonstrate that human ALOX15 and ALOX15B catalyze the first two steps in the PD$_{n\text{-}3\ DPA}$ biosynthetic pathway yielding an allylic epoxide. Using total organic synthesis, we established the absolute stereochemistry of this epoxide as 16*S*,17*S*-epoxy-7*Z*,10*Z*,12*E*,14*E*,19*Z*-docosapentaenoic acid. Furthermore, using this synthetic material we found that 16*S*,17*S*-ePD$_{n\text{-}3\ DPA}$ was converted to PD1$_{n\text{-}3\ DPA}$ and PD2$_{n\text{-}3\ DPA}$ by different epoxide hydrolase enzymes, where in human cells EPHX2 was found to catalyze the conversion of 16*S*,17*S*-ePD$_{n\text{-}3\ DPA}$ to PD2$_{n\text{-}3\ DPA}$ (Figure 7). Of note, we also found that LTA_4H was not involved in the biosynthesis of PD1$_{n\text{-}3\ DPA}$.

In summation, the present findings establish the identity of enzymes involved in the PD$_{n\text{-}3\ DPA}$ biosynthetic pathway, as well as the complete stereochemistry of the key intermediate in this pathway (Figure 7F). They also demonstrate that the PD$_{n\text{-}3\ DPA}$ pathway plays a role in the differentiation of monocytes to macrophages, where inhibition of 15-lipoxygenase activity during monocyte-to-macrophage differentiation results in impaired monocyte-derived macrophage responses that are rescued by addition of PD$_{n\text{-}3\ DPA}$. Thus, these findings establish candidates in understanding the etiopathology of inflammatory diseases as well as targets for patient stratification and essential fatty acid supplementation.

SIGNIFICANCE

Macrophages are central players in controlling the body's response to both sterile injury and infections. During the course of inflammation monocytes are recruited to the site of injury and/or infections, where they differentiate to macrophages. The phenotype displayed by these macrophages dictates whether the cells promote the termination of inflammation or lead to chronicity, with the mechanisms that control the monocyte-to-macrophage differentiation process remaining of interest. In the present study, we found that inhibition of ALOX15 activity during monocyte-to-macrophage differentiation reduced PD$_{n\text{-}3\ DPA}$ production, altered human macrophage phenotype and their ability to clear dead cells, a key step in the termination of inflammation. Incubation of these cells with components of the PD$_{n\text{-}3\ DPA}$ biosynthetic pathway rectified these responses. We established the identity and complete stereochemistry of 16*S*,17*S*-ePD$_{n\text{-}3\ DPA}$, a key intermediate in this pathway, and the role of human 15-lipoxygenases in producing this intermediate. We also provide evidence for a role of epoxide hydrolase enzymes in catalyzing the conversion of this intermediate to the pro-resolving mediators PD1$_{n\text{-}3\ DPA}$ and PD2$_{n\text{-}3\ DPA}$ in human monocytes. Here we found that EPHX2 selectively catalyzes the conversion of 16*S*,17*S*-ePD$_{n\text{-}3\ DPA}$ to PD2$_{n\text{-}3\ DPA}$. Together these results establish the PD$_{n\text{-}3\ DPA}$ pathway in human monocytes and the contribution of this pathway in monocyte-to-macrophage differentiation.

(D and E) Mice were treated as in (A–C), and on day 7 administered fluorescently labeled (D) apoptotic cells (6×10^6 cells/mouse) or (E) *E. coli* (10^6 CFU/mouse) via an intraperitoneal injection. Peritoneal cells were collected after 1 hr and phagocytosis was assessed in (D) $CD64^+$ large peritoneal macrophages (left panel) and small peritoneal macrophages (right panel), and (E) total $CD64^+$ macrophage population. Results are mean ± SEM. n = 8 mice per group for (D) and n = 4 mice per group for (E). *$p < 0.05$.
(F) Structures are illustrated in most likely configurations based on biosynthetic evidence. The stereochemistries for PD1$_{n\text{-}3\ DPA}$ and 16*S*,17*S*-PD$_{n\text{-}3\ DPA}$ are established (Aursnes et al., 2015; Aursnes et al., 2014; Dalli et al., 2013a).
Related to Figures S5.

STAR★METHODS

Detailed methods are provided in the online version of this paper and include the following:

- KEY RESOURCES TABLE
- CONTACT FOR REAGENT AND RESOURCE SHARING
- EXPERIMENTAL MODEL AND SUBJECT DETAILS
  - Animal Studies
  - Human Primary Cells
- METHOD DETAILS
  - Monocyte Incubations
  - Enzyme Incubations
  - Flow Cytometry
  - Macrophage Incubations
  - Lipid Mediator Profiling
  - Total Organic Synthesis of 16*S*,17*S*-ePD$_{\text{n-3 DPA}}$
- QUANTIFICATION AND STATISTICAL ANALYSIS
- DATA AND SOFTWARE AVAILABILITY

SUPPLEMENTAL INFORMATION

Supplemental Information includes five figures and three tables and can be found with this article online at https://doi.org/10.1016/j.chembiol.2018.04.017.

ACKNOWLEDGMENTS

The School of Pharmacy, University of Oslo is gratefully acknowledged for a Ph.D. scholarship to K.G.P. The Norwegian Research Council (FRIPRO/FRINATEK 230470) is gratefully acknowledged for funding to T.V.H. J.D. received funding from the European Research Council (ERC) under the European Union's Horizon 2020 research and innovation program (grant no. 677542) and the Barts Charity (grant no. MGU0343). J.D. is also supported by a Sir Henry Dale Fellowship jointly funded by the Wellcome Trust and the Royal Society (grant no. 107613/Z/15/Z). This work is also supported by a Wellcome Trust Infrastructure (grant no. 101604/Z/13/Z).

AUTHOR CONTRIBUTIONS

K.P., P.R.S., K.G.P., R.A.C., R.D.M., S.A.-W., R.M.M., F.M., and J.D. carried out the experiments and analyzed the data. A.V., T.V.H., and J.D. designed the experiments. All authors contributed to the preparation of the paper. J.D. conceived the overall research plan.

DECLARATION OF INTERESTS

J.D. is an inventor on a patent detailing the structure and actions of mediators described in this manuscript (PCT/US2014/037969). Remaining authors declare no competing interests.

Received: September 12, 2017
Revised: January 31, 2018
Accepted: April 25, 2018
Published: May 24, 2018

REFERENCES

Aursnes, M., Tungen, J.E., Colas, R.A., Vlasakov, I., Dalli, J., Serhan, C.N., and Hansen, T.V. (2015). Synthesis of the 16S,17S-epoxyprotectin intermediate in the biosynthesis of protectins by human macrophages. J. Nat. Prod. *78*, 2924–2931.

Aursnes, M., Tungen, J.E., Vik, A., Colas, R., Cheng, C.Y., Dalli, J., Serhan, C.N., and Hansen, T.V. (2014). Total synthesis of the lipid mediator PD1n-3 DPA: configurational assignments and anti-inflammatory and pro-resolving actions. J. Nat. Prod. *77*, 910–916.

Borgeat, P., Fruteau de Laclos, B., Picard, S., Drapeau, J., Vallerand, P., and Corey, E.J. (1982). Studies on the mechanism of formation of the 5S, 12S-dihydroxy-6,8,10,14(E, Z,E,Z)-icosatetraenoic acid in leukocytes. Prostaglandins *23*, 713–724.

Bystrom, J., Evans, I., Newson, J., Stables, M., Toor, I., van Rooijen, N., Crawford, M., Colville-Nash, P., Farrow, S., and Gilroy, D.W. (2008). Resolution-phase macrophages possess a unique inflammatory phenotype that is controlled by cAMP. Blood *112*, 4117–4127.

Chiang, N., and Serhan, C.N. (2017). Structural elucidation and physiologic functions of specialized pro-resolving mediators and their receptors. Mol. Aspects Med. *58*, 114–129.

Claria, J., Lee, M.H., and Serhan, C.N. (1996). Aspirin-triggered lipoxins (15-epi-LX) are generated by the human lung adenocarcinoma cell line (A549)-neutrophil interactions and are potent inhibitors of cell proliferation. Mol. Med. *2*, 583–596.

Colas, R.A., Dalli, J., Chiang, N., Vlasakov, I., Sanger, J.M., Riley, I.R., and Serhan, C.N. (2016). Identification and actions of the maresin 1 metabolome in infectious inflammation. J. Immunol. *197*, 4444–4452.

Dalli, J., Chiang, N., and Serhan, C.N. (2015). Elucidation of novel 13-series resolvins that increase with atorvastatin and clear infections. Nat. Med. *21*, 1071–1075.

Dalli, J., Colas, R.A., and Serhan, C.N. (2013a). Novel n-3 immunoresolvents: structures and actions. Sci. Rep. *3*, 1940.

Dalli, J., and Serhan, C. (2016). Macrophage proresolving mediators - the when and where. Microbiol. Spectr. *4*.

Dalli, J., and Serhan, C.N. (2012). Specific lipid mediator signatures of human phagocytes: microparticles stimulate macrophage efferocytosis and pro-resolving mediators. Blood *120*, e60–e72.

Dalli, J., Winkler, J.W., Colas, R.A., Arnardottir, H., Cheng, C.Y., Chiang, N., Petasis, N.A., and Serhan, C.N. (2013b). Resolvin D3 and aspirin-triggered resolvin D3 are potent immunoresolvents. Chem. Biol. *20*, 188–201.

Dalli, J., Zhu, M., Vlasenko, N.A., Deng, B., Haeggstrom, J.Z., Petasis, N.A., and Serhan, C.N. (2013c). The novel 13S,14S-epoxy-maresin is converted by human macrophages to maresin 1 (MaR1), inhibits leukotriene A4 hydrolase (LTA4H), and shifts macrophage phenotype. FASEB J. *27*, 2573–2583.

Deng, B., Wang, C.W., Arnardottir, H.H., Li, Y., Cheng, C.Y., Dalli, J., and Serhan, C.N. (2014). Maresin biosynthesis and identification of maresin 2, a new anti-inflammatory and pro-resolving mediator from human macrophages. PLoS One *9*, e102362.

Dona, M., Fredman, G., Schwab, J.M., Chiang, N., Arita, M., Goodarzi, A., Cheng, G., von Andrian, U.H., and Serhan, C.N. (2008). Resolvin E1, an EPA-derived mediator in whole blood, selectively counterregulates leukocytes and platelets. Blood *112*, 848–855.

El Kebir, D., Gjorstrup, P., and Filep, J.G. (2012). Resolvin E1 promotes phagocytosis-induced neutrophil apoptosis and accelerates resolution of pulmonary inflammation. Proc. Natl. Acad. Sci. USA *109*, 14983–14988.

Fredman, G., Hellmann, J., Proto, J.D., Kuriakose, G., Colas, R.A., Dorweiler, B., Connolly, E.S., Solomon, R., Jones, D.M., Heyer, E.J., et al. (2016). An imbalance between specialized pro-resolving lipid mediators and pro-inflammatory leukotrienes promotes instability of atherosclerotic plaques. Nat. Commun. *7*, 12859.

Fredman, G., Ozcan, L., Spolitu, S., Hellmann, J., Spite, M., Backs, J., and Tabas, I. (2014). Resolvin D1 limits 5-lipoxygenase nuclear localization and leukotriene B4 synthesis by inhibiting a calcium-activated kinase pathway. Proc. Natl. Acad. Sci. USA *111*, 14530–14535.

Gobbetti, T., Dalli, J., Colas, R.A., Federici Canova, D., Aursnes, M., Bonnet, D., Alric, L., Vergnolle, N., Deraison, C., Hansen, T.V., et al. (2017). Protectin D1n-3 DPA and resolvin D5n-3 DPA are effectors of intestinal protection. Proc. Natl. Acad. Sci. USA *114*, 3963–3968.

Gordon, S., and Mantovani, A. (2011). Diversity and plasticity of mononuclear phagocytes. Eur. J. Immunol. *41*, 2470–2472.

Isobe, Y., Arita, M., Matsueda, S., Iwamoto, R., Fujihara, T., Nakanishi, H., Taguchi, R., Masuda, K., Sasaki, K., Urabe, D., et al. (2012). Identification and structure determination of novel anti-inflammatory mediator resolvin E3, 17,18-dihydroxyeicosapentaenoic acid. J. Biol. Chem. *287*, 10525–10534.

Janes, K.A., and Yaffe, M.B. (2006). Data-driven modelling of signal-transduction networks. Nat. Rev. Mol. Cell Biol. *7*, 820–828.

Kopf, M., Schneider, C., and Nobs, S.P. (2015). The development and function of lung-resident macrophages and dendritic cells. Nat. Immunol. *16*, 36–44.

Kumar, D.A., and Meshram, H.M. (2013). Concise stereoselective total synthesis of (+)-mueggelone. Synth. Commun. *43*, 1145–1154.

Maryanoff, B.E., and Reitz, A.B. (1989). The Wittig olefination reaction and modifications involving phosphoryl-stabilized carbanions. Stereochemistry, mechanism, and selected synthetic aspects. Chem. Rev. *89*, 863–927.

Morris, T., Stables, M., Colville-Nash, P., Newson, J., Bellingan, G., de Souza, P.M., and Gilroy, D.W. (2010). Dichotomy in duration and severity of acute inflammatory responses in humans arising from differentially expressed proresolution pathways. Proc. Natl. Acad. Sci. USA *107*, 8842–8847.

Mottola, G., Chatterjee, A., Wu, B., Chen, M., and Conte, M.S. (2017). Aspirin-triggered resolvin D1 attenuates PDGF-induced vascular smooth muscle cell migration via the cyclic adenosine monophosphate/protein kinase A (cAMP/PKA) pathway. PLoS One *12*, e0174936.

Newson, J., Stables, M., Karra, E., Arce-Vargas, F., Quezada, S., Motwani, M., Mack, M., Yona, S., Audzevich, T., and Gilroy, D.W. (2014). Resolution of acute inflammation bridges the gap between innate and adaptive immunity. Blood *124*, 1748–1764.

Ohishi, N., Izumi, T., Minami, M., Kitamura, S., Seyama, Y., Ohkawa, S., Terao, S., Yotsumoto, H., Takaku, F., and Shimizu, T. (1987). Leukotriene A4 hydrolase in the human lung. Inactivation of the enzyme with leukotriene A4 isomers. J. Biol. Chem. *262*, 10200–10205.

Petasis, N.A., Yang, R., Winkler, J.W., Zhu, M., Uddin, J., Bazan, N.G., and Serhan, C.N. (2012). Stereocontrolled total synthesis of neuroprotectin D1/protectin D1 and its aspirin-triggered stereoisomer. Tetrahedron Lett. *53*, 1695–1698.

Pierdomenico, A.M., Recchiuti, A., Simiele, F., Codagnone, M., Mari, V.C., Davi, G., and Romano, M. (2015). MicroRNA-181b regulates ALX/FPR2 receptor expression and proresolution signaling in human macrophages. J. Biol. Chem. *290*, 3592–3600.

Primdahl, K.G., Tungen, J.E., De Souza, P.R.S., Colas, R.A., Dalli, J., Hansen, T.V., and Vik, A. (2017). Stereocontrolled synthesis and investigation of the biosynthetic transformations of 16(S),17(S)-epoxy-PDn-3 DPA. Org. Biomol. Chem. *15*, 8606–8613.

Qiu, H., Gabrielsen, A., Agardh, H.E., Wan, M., Wetterholm, A., Wong, C.H., Hedin, U., Swedenborg, J., Hansson, G.K., Samuelsson, B., et al. (2006). Expression of 5-lipoxygenase and leukotriene A4 hydrolase in human atherosclerotic lesions correlates with symptoms of plaque instability. Proc. Natl. Acad. Sci. USA *103*, 8161–8166.

Radmark, O., Malmsten, C., Samuelsson, B., Clark, D.A., Goto, G., Marfat, A., and Corey, E.J. (1980). Leukotriene A: stereochemistry and enzymatic conversion to leukotriene B. Biochem. Biophys. Res. Commun. *92*, 954–961.

Rudberg, P.C., Tholander, F., Thunnissen, M.M., Samuelsson, B., and Haeggstrom, J.Z. (2002). Leukotriene A4 hydrolase: selective abrogation of leukotriene B4 formation by mutation of aspartic acid 375. Proc. Natl. Acad. Sci. USA *99*, 4215–4220.

Sendobry, S.M., Cornicelli, J.A., Welch, K., Bocan, T., Tait, B., Trivedi, B.K., Colbry, N., Dyer, R.D., Feinmark, S.J., and Daugherty, A. (1997). Attenuation of diet-induced atherosclerosis in rabbits with a highly selective 15-lipoxygenase inhibitor lacking significant antioxidant properties. Br. J. Pharmacol. *120*, 1199–1206.

Serhan, C.N., Clish, C.B., Brannon, J., Colgan, S.P., Chiang, N., and Gronert, K. (2000). Novel functional sets of lipid-derived mediators with antiinflammatory actions generated from omega-3 fatty acids via cyclooxygenase 2-nonsteroidal antiinflammatory drugs and transcellular processing. J. Exp. Med. *192*, 1197–1204.

Serhan, C.N., Hamberg, M., and Samuelsson, B. (1984). Trihydroxytetraenes: a novel series of compounds formed from arachidonic acid in human leukocytes. Biochem. Biophys. Res. Commun. *118*, 943–949.

Serhan, C.N., Yang, R., Martinod, K., Kasuga, K., Pillai, P.S., Porter, T.F., Oh, S.F., and Spite, M. (2009). Maresins: novel macrophage mediators with potent antiinflammatory and proresolving actions. J. Exp. Med. *206*, 15–23.

Sica, A., Saccani, A., Bottazzi, B., Bernasconi, S., Allavena, P., Gaetano, B., Fei, F., LaRosa, G., Scotton, C., Balkwill, F., et al. (2000). Defective expression of the monocyte chemotactic protein-1 receptor CCR2 in macrophages associated with human ovarian carcinoma. J. Immunol. *164*, 733–738.

Tungen, J.E., Aursnes, M., Dalli, J., Arnardottir, H., Serhan, C.N., and Hansen, T.V. (2014a). Total synthesis of the anti-inflammatory and pro-resolving lipid mediator MaR1n-3 DPA utilizing an sp(3) -sp(3) Negishi cross-coupling reaction. Chem. Eur. J. *20*, 14575–14578.

Tungen, J.E., Aursnes, M., Vik, A., Ramon, S., Colas, R.A., Dalli, J., Serhan, C.N., and Hansen, T.V. (2014b). Synthesis and anti-inflammatory and pro-resolving activities of 22-OH-PD1, a monohydroxylated metabolite of protectin D1. J. Nat. Prod. *77*, 2241–2247.

Winkler, J.W., Orr, S.K., Dalli, J., Cheng, C.Y., Sanger, J.M., Chiang, N., Petasis, N.A., and Serhan, C.N. (2016). Resolvin D4 stereoassignment and its novel actions in host protection and bacterial clearance. Sci. Rep. *6*, 18972.

STAR★METHODS

KEY RESOURCES TABLE

REAGENT or RESOURCE	SOURCE	IDENTIFIER
Antibodies		
APC/Cy7 anti-human CD 14	Biolegend	Clone 63D3;<br>Cat # 367107
PE-Cy7 anti-human CD 32	eBiosciences	Clone 6C4;<br>Cat # 25-0329-41
PE-Cy5 anti-CD 64	Abcam	Clone 10.1;<br>Cat # ab192338
Alexa Fluor 405 anti-ICAM-1	Novus	Clone 1A29;<br>Cat # NBP2-22541
Alexa Fluor anti-human 488 CD 68	Biolegend	Clone Y1/82A;<br>Cat # 333811
Brilliant Violet anti-human 650 CD 80	Biolegend	Clone 2D10;<br>Cat # 305227
PerCP-Cy5.5 anti-human CD 206	Biolegend	Clone 15-2;<br>Cat # 321121
PE-CF 594 mouse anti-human CD 163	BD Biosciences	Clone GHI/61;<br>Cat # 562670
Monoclonal anti-ALOX15B	Sigma	Clone 4A7<br>Cat # SAB1402114-100UG
EPXH2 antibody [2F2]	Gene Tex	Cat # GTX84567
Alexa Fluor 488 Goat anti-mouse IgG (H + L)	Invitrogen	Cat # A11029
Alexa Fluor 647 15-Lipoxygenase 1 rabbit polyclonal	Bioss	Cat # bs-6505R-A647
APC-Cy7 anti-mouse CD 64	Biolegend	Clone X54-5/7.1<br>Cat # 139303
PE anti-mouse CD 64 (FCγRI)	Biolegend	Clone X54-5/7.1<br>Cat # 139303
PE-Cy5 anti-mouse/human CD 11b	Biolegend	Clone M1/70<br>Cat # 101210
Brilliant Violet 650 rat anti-mouse I-A/I-E (MHC II)	Biolegend	Clone M5/114.15.2<br>Cat # 107641
APC-Cy7 rat anti-mouse F4/80	Biolegend	Clone BM8<br>Cat # 123117
BV785 hamster anti-mouse CD 11c	Biolegend	Clone N418<br>Cat # 117336
PerCP-eFluor710 rat anti-mouse TIM-4	eBiosciences	Clone 54 (RMT4-54)<br>Cat # 46-5866-28
Alexa Fluor 488 COX 2	Cell Signaling Technologies	Clone (D5H5) XP ®<br>Cat # 13596S
PE-Dazzle 594 rat anti-mouse IL-10	Biolegend	Clone JES5-16E3<br>Cat # 505034
Brilliant Violet mouse anti-mouse 421 TGF-β1	Biolegend	Clone TW7-16B4<br>Cat # 141407
PE sheep anti-mouse Arginase 1	R&D	Cat # IC5868P
Alexa Fluor 647 rabbit- anti-human iNOS	Novus	Clone 4E5<br>Cat # NBP2-22119AF647
TruStain Fc-blocking IgG (anti-mouse CD16/32)	Biolegend	Clone 93<br>Cat # 101310

(*Continued on next page*)

Continued		
REAGENT or RESOURCE	SOURCE	IDENTIFIER
Bacterial and Virus Strains		
Escherichia coli		Strain O6:K2:H1
Biological Samples		
Leukocyte cones	GBS Re NHS Blood/Transplant	Cat # NC24
Chemicals, Peptides, and Recombinant Proteins		
Ethanol, Absolute (200 proof), Mol Biology grade, Dnase, Rnase, Protease-free	Fisher Scientific Uk Ltd	Cat # 10644795
Methanol, Optima(TM) LC/MS grade	Fisher Scientific Uk Ltd	Cat # 10767665
Acetic acid	Fluka	Cat # 07692-1L-F
Methyl formate, 98% for spectroscopy	Fisher Scientific Uk Ltd	Cat # 10414315
n-Hexane; For HPLC; 97+%; Acros Organics	Fisher Scientific Uk Ltd	Cat # 11934421
Deuterium-labelled 5S-HETE	Cayman Chemicals	Cat # 10007276
Deuterium-labelled Leukotriene (LT) B_4	Cayman Chemicals	Cat #: 320110
Deuterium-labelled Lipoxin (LX) A_4	Cayman Chemicals	Cat #: 10007737
Deuterium-labelled Resolvin (Rv) D2	Cayman Chemicals	Cat #: 11184
Deuterium-labelled prostaglandin (PG) E_2	Cayman Chemicals	Cat #: 314010
PGD_2	Cayman Chemicals	Cat #: 10007202
PGE_2	Cayman Chemicals	Cat #: 10007211
PGF_{2a}	Cayman Chemicals	Cat # 16010
Thromboxane B_2	Cayman Chemicals	Cat #: 10007237
LTB_4	Cayman Chemicals	Cat #: 10007240
20-OH-LTB_4	Cayman Chemicals	Cat # 20190
LXA_4	Cayman Chemicals	Cat #: 10007271
LXB_4	Cayman Chemicals	Cat #: 90420
5S,12S-diHETE	In-house biogenic synthesis (J Dalli)	(Borgeat et al., 1982)
5S,15S-diHETE	Cayman Chemicals	Cat #: 35280
15-epi-LXA_4	Cayman Chemicals	Cat #: 90415
15-epi-LXB_4	Custom Synthesis (Dr Charles Serhan, Harvard Medical School)	(Claria et al., 1996)
RvE1	Cayman Chemicals	Cat #: 10007848
RvE2	Custom synthesis	(Serhan et al., 2000)
RvE3	Custom synthesis	(Isobe et al., 2012)
RvD1	Cayman Chemicals	Cat #: 10012554
RvD2	Cayman Chemicals	Cat #: 10007279
RvD3	Cayman Chemicals	Cat #: 13834
RvD4	Custom Synthesis (Dr Charles Serhan, Harvard Medical School)	(Winkler et al., 2016)
RvD5	Cayman Chemicals	Cat #: 10007280
RvD6	In-house biogenic synthesis (J Dalli)	N/A
17R-RvD1	Cayman Chemicals	Cat #: 13060
17R-RvD3	Custom Synthesis (Dr Charles Serhan, Harvard Medical School)	Dalli et al., 2013b
Maresin (MaR) 1	Cayman Chemicals	Cat #: 10878
MaR2	Cayman Chemicals	Cat #: 16369
4S,14S-diHDHA	In-house biogenic synthesis (J Dalli)	(Serhan et al., 2009)
7S,14S-diHDHA	In-house biogenic synthesis (J Dalli)	(Serhan et al., 2009)
22-OH-MaR1	In-house biogenic synthesis (J Dalli)	(Colas et al., 2016)
14-oxo-MaR1	In-house biogenic synthesis (J Dalli)	(Colas et al., 2016)
Protectin (PD)1	Custom Synthesis (Dr Charles Serhan, Harvard Medical School)	(Petasis et al., 2012)

(*Continued on next page*)

Continued

REAGENT or RESOURCE	SOURCE	IDENTIFIER
10S,17S-diHDHA	Custom Synthesis (Dr Charles Serhan, Harvard Medical School)	(Petasis et al., 2012)
22-OH-PD1	Custom Synthesis (Dr Trond V. Hansen, University of Oslo)	(Tungen et al., 2014b)
n-3 DPA	Cayman Chemicals	Item № 21907
$RvD1_{n\text{-}3\ DPA}$	In-house biogenic synthesis (J Dalli)	(Dalli et al., 2013a)
$RvD2_{n\text{-}3\ DPA}$	In-house biogenic synthesis (J Dalli)	Dalli et al. (2013a)
$RvD5_{n\text{-}3\ DPA}$	In-house biogenic synthesis (J Dalli)	Dalli et al. (2013a)
$MaR1_{n\text{-}3\ DPA}$	Custom Synthesis (Dr Trond V. Hansen, University of Oslo)	(Tungen et al., 2014a)
7S, 14S-diHDPA	In-house biogenic synthesis (J Dalli)	Dalli et al. (2013a)
$PD1_{n\text{-}3\ DPA}$	Custom Synthesis (Dr Trond V. Hansen, University of Oslo)	Aursnes et al. (2014)
$PD2_{n\text{-}3\ DPA}$	In-house biogenic synthesis (J Dalli)	Dalli et al. (2013a)
16S, 17S-$ePD_{n\text{-}3\ DPA}$	Custom Synthesis (Dr Trond V. Hansen, University of Oslo)	This paper
10S,17S-diHDPA	In-house biogenic synthesis (J Dalli)	Dalli et al. (2013a)
Δ15trans-$PD1_{n\text{-}3\ DPA}$	In-house biogenic synthesis (J Dalli)	This paper
10epi-Δ15trans-$PD1_{n\text{-}3\ DPA}$	In-house biogenic synthesis (J Dalli)	This paper
17R-PD1	Custom Synthesis (Dr Charles Serhan, Harvard Medical School)	Petasis et al. (2012)
RvT1	In-house biogenic synthesis (J Dalli)	(Dalli et al., 2015)
RvT2	In-house biogenic synthesis (J Dalli)	Dalli et al. (2015)
RvT3	In-house biogenic synthesis (J Dalli)	Dalli et al. (2015)
RvT4	In-house biogenic synthesis (J Dalli)	Dalli et al. (2015)
Histopaque – 1077	Sigma	Cat # 10771-100ML
Dulbecco's phosphate buffer saline with $MgCl_2$ and $CaCl_2$ ($PBS^{+/+}$)	Sigma	Cat # D8662
Dulbecco's phosphate buffer saline without $MgCl_2$ and $CaCl_2$ ($PBS^{-/-}$)	Sigma	Cat # D8537
RPMI-1640	Sigma	Cat # R8758
RPMI 1640 Medium, no glutamine, no phenol red	Gibco/Life Technologies	Cat # 32404014
Recombinant Human M-CSF	R&D	Cat # 216-MC-025
PD146176	Cambridge Bioscience	Cat # 10010518
Human serum type AB (male)	Sigma	Cat # H4522-100ML
Penicillin-Streptomycin	Sigma	Cat # P4333
Fetal bovine serum (FBS)	Gibco/Life Technologies	Cat # 105000-64
DMSO, cell culture reagent	ChemCruz	Cat # sc-358801
Bovine serum albumin (BSA)	Sigma	Cat # A9418
EDTA	Invitrogen	Cat # 15575-038
PKH26 Red Fluorescent Cell Linker kit	Sigma	Cat # PKH26GL-1KT
PKH67 Green Fluorescent Cell Linker kit	Sigma	Cat # PKH67GL-1KT
Trypan Blue	Sigma	Cat # T8154
Polybrene Transfection Reagent	Millipore, UK Ltd	Cat # TR-1003-G
Recombinant Human GM-CSF	Biolegend	Cat # 572902
Human recombinant INF-γ	Biolegend	Cat # 570206
Human recombinant IL-4	Biolegend	Cat # 574002
Lipopolysaccharides	Sigma	Cat # L2630

(*Continued on next page*)

Continued

REAGENT or RESOURCE	SOURCE	IDENTIFIER
ALOX15 Human, 4 unique 29mer shRNA constructs in lentiviral GFP vector (Gene ID = 246). 5μg purified plasmid DNA per construct	OriGENE	TL314822
MISSION shRNA Bacterial Clone ALXO15B	Sigma	Cat #s SHCLNG - TRCN0000432221, TRCN0000056583, TRCN0000056584, TRCN0000056585, TRCN0000056586
MISSION shRNA Bacterial Glycerol Stock EPHX2	Sigma	Cat #s SHCLNG - TRCN0000050553, TRCN0000050554, TRCN0000050555, TRCN0000050556, TRCN0000050557
MISSION® pLKO.1-puro Empty Vector Control Plasmid DNA	Sigma	Cat # SHC001
Trizma hydrochloride	Sigma	Cat # T3253
hr-ALOX15	Novus Biologicals	Cat # H00000246-P01
hr-ALOX15B	Cayman Chemicals	Cat # 10011263-100ug-CAY
hr-EPHX2	Cayman Chemicals	Cat # 10011669
AUDA	Sigma	Cat # SML0177
4% PFA solution	Affymetrix	Cat # 19943
Permeabilization buffer (10X)	eBiosciences	Cat # 00-8333-56
Fixation/Permeabilization Concentrate	eBiosciences	Cat # 00-5123-43
Fixation/Permeabilization Diluent	eBiosciences	Cat # 00-5223-56
Hanks Balanced Salt solution	Sigma	Cat # H6648
γ-Globulins from human blood (FC block)	Sigma	Cat # G4386-5G
LIVE/DEAD BacLight Bacterial Viability dye	Molecular Probes, Life Technologies	Cat # B35000
Critical Commercial Assays		
APC Annexin V Apoptosis Detection Kit with PI	Biolegend	Cat # 640932
EasySep™ Human Monocyte Isolation Kit	StemCell Technologies	Cat # 19319
Experimental Models: Organisms/Strains		
Mus musculus, NCBI Taxonomy ID:10090, C57BL/6/FVB	Charles River	JAX™ C57BL/6J
Mus musculus, NCBI Taxonomy ID:10090, C57BL/6/FVB 12/15-LOX KO	The Jackson Laboratory	B6.129S2-*Alox15*^{tm1Fun}/J Stock # 002778
Experimental Models: Cell lines		
HL-60	ATCC	Cat # CCL-240
Software and Algorithms		
FlowJo software	Tree Star, Ashland, OR	https://www.flowjo.com/solutions/flowjo/downloads
GraphPad Prism 6.0f	GraphPad Software, CA	https://www.graphpad.com/support/prism-6-updates/
SIMCA 14.1 software	Umetrics, Umea, Sweden	https://umetrics.com/kb/simca-141
IDEAS® (Image Data Exploration and Analysis Software, Version 6.0)	Amnis®, EMD Millipore	http://www.merckmillipore.com/GB/en/life-science-research/cell-analysis-flow-cytometry/amnis-imaging-flow-cytometers/analysis-acquisition-software/ideas-software/qe2b.qB.Oq8AAAFLQM8Jx34R,nav
Other		
Isolute 500 mg / 3ml C18 SPE column	Biotage, Sweden	Cat # 220-0050-B
Poroshell 120 EC-18 4.6 mm ×100 mm × 2.7 μm reversed phase column	Agilent, USA	N/A
LSR Fortessa cell analyser	BD Biosciences, UK	N/A
Extra-Hera	Biotage, Sweden	N/A

(*Continued on next page*)

Continued

REAGENT or RESOURCE	SOURCE	IDENTIFIER
TurboVap LV	Biotage, Sweden	N/A
Qtrap 5500/6500	AB Sciex	N/A
Shimadzu SIL-20AC auto-injector	Shimadzu Corp.	N/A
LC-20AD Binary pump	Shimadzu Corp.	N/A
FLUOstar Omega microplate reader	BMG Labtech	N/A
ImageStream X MK2 Imaging Flow Cytometer	Amnis®, EMD Millipore	N/A

CONTACT FOR REAGENT AND RESOURCE SHARING

Further information and requests for resources and reagents should be directed to and will be fulfilled by the Lead Contact, Jesmond Dalli (j.dalli@qmul.ac.uk).

EXPERIMENTAL MODEL AND SUBJECT DETAILS

Animal Studies

Healthy 6-11 week old male C57/Black6 wild type mice (Charles River) and ALOX15$^{-/-}$ (The Jackson Laboratory) mice were used in the reported studies. The experiments strictly adhered to UK Home Office regulations (Guidance on the Operation of Animals, Scientific Procedures Act, 1986) and Laboratory Animal Science Association (LASA) Guidelines (Guiding Principles on Good Practice for Animal Welfare and Ethical Review Bodies, 3rd Edition, 2015). Animals were kept on a 12 h light dark cycle, with lights turned on at 7:00 h and lights turned off at 19:00h under specific pathogen free housing and had access to food and water *ad libitum*. Sample size was based on the statistical analysis of previous experiments and no mice were excluded. Animals were randomly assigned to control and experimental groups. The investigators were not blinded to group assignments.

Human Primary Cells

Healthy human peripheral blood mononuclear cells (PBMCs) were isolated from leukocyte cones obtained from the NHS Blood and Transplant bank and experiments were conducted in accordance with a protocol approved by Queen Mary Research Ethics Committee (QMREC 2014:61) and in accordance with the Helsinki declaration. Informed consent was obtained from all volunteers. Given that cones were obtained from the NHS Blood and Transplant bank and volunteers were unidentified, no information was available on sex, age, their involvement in previous experiments or if they were drug or test naïve. Primary cells were incubated for at 37°C at 5 % CO_2. The HL60 cell line was established in a female donor.

METHOD DETAILS

Monocyte Incubations

Human PBMCs were isolated from healthy human volunteers purchased from the NHS Blood and Transplant bank and experiments conducted in Queen Mary Research Ethics Committee approval (QMREC 2014:61) and the Helsinki declaration. Here blood cones were used and PBMCs were isolated by density centrifugation where cells were layered on to Histopaque - 1077 (Sigma) and centrifuged for 30 minutes at 400 x g at room temperature (RT). Macrophages were prepared using published protocols (Dalli and Serhan, 2012) where PBMCs were plated into 10 cm tissue culture plates and incubated at 37°C for 30 min in PBS$^{+/+}$. Non-adherent cells were then removed by adding PBS$^{-/-}$ and washed vigorously. Adherent cells (3×10^7 cells/incubation) were then incubated with vehicle (PBS+0.1% ethanol), 1 nM 16*S*,17*S*-ePD$_{n-3\ DPA}$ or 1 nM PD1$_{n-3\ DPA}$ in RPMI 1640 (Sigma) for 30 minutes at 37°C. These were then incubated with either vehicle or ALOX15 inhibitor (10 μM PD146176 (Sendobry et al., 1997); Cambridge Bioscience) in RPMI 1640 containing 10% human serum, 1% Penicillin-Streptomycin and 20 ng/mL M-CSF. The cells were incubated for 3 days at 37°C at 5% CO_2 and the media together with mediators and inhibitors were added as above and the cells were incubated a further 4 days. For lipid mediator profiling of cell supernatant, RPMI 1640 Medium (no glutamine) with no phenol red was used. Cell supernatants were collected and placed in two volumes of methanol containing deuterium labelled internal standards (500 pg of d$_4$-Prostaglandin (PG)E$_2$, d$_5$-LXA$_4$, d$_4$-RvD2, d$_4$-Leukotriene (LT)B$_4$ and d$_8$-5*S*-Hydroxy-eicosatetraneoic acid; Cayman Chemicals). These were stored at -20°C until extraction (see below). In select experiments to obtain M1 macrophages adherent cells were cultured with GM-CSF (20ng/mL) for 6 days then with LPS (1ng/mL) and interferon-γ (20ng/mL) for 24h. To obtain M2 macrophages adherent cells where incubated with M-CSF (20ng/mL) for 7 days then with IL-4 (20ng/mL) for 48 h. In all cases the purity of the cell preparations ranged between 90-95%.

For human monocyte incubations we isolated monocytes from PBMCs using the EasySep™ Human Monocyte Isolation Kit, following manufacturer's instruction where the purity of the resultant cell population was of ~95%. Human monocytes (1×10^8 cells/mL) were incubated with n-3 DPA (10μM) and *Escherichia coli* (5×10^9 CFU/mL) in PBS at 37°C. Incubations were quenched

using excess acidified methanol (apparent pH ~3) containing deuterium labelled d_4-LTB_4 and products were extracted as detailed below.

In select experiments monocytes (1x10^8 cells/ml) were incubated with vehicle (PBS + 0.01% DMSO) or 12-[[(tricyclo[3.3.1.13,7] dec-1-ylamino)carbonyl]amino]-dodecanoic acid (AUDA; 25μM) for 20 min at RT. Cells were then incubated with either vehicle (PBS + 0.1%EtOH) or 16*S*,17*S*-e-$PD_{n\text{-}3\ DPA}$ (10nM) for 15 min. Incubations were then quenched and products identified and profiled using LM profiling as detailed below.

Monocytes (1.5x10^6 cells/ml) were suspended in phenol red free RPMI 1640 containing 10% human serum, 8μg/ml of polybrene and 50ng/ml of shRNA to ALOX15B or CT shRNA. The cell suspension was then centrifuged at 1000 x g for 90 min at 4°C. Cells were then plated and incubated for a further 12h at 37°C. Monocytes were then detached after 5 minutes incubation in 2mM EDTA in PBS$^{-/-}$, and the expression of ALOX15B was evaluated using flow cytometry. Cells were also suspended in PBS containing 0.1% human serum and incubated with *E. coli* (1:50 monocytes to bacteria) for 45 min at 37°C. Incubations were quenched using two volumes of ice-cold methanol and products profiled using lipid mediator profiling.

In separate experiments monocytes were plated in 6 well plates and incubated with phenol red free RPMI 1640 containing 10% human serum, 8μg/ml of polybrene and 2μg/ml of shRNA to ALOX15, EPHX2 or CT shRNA. The cell incubations were gently mixed for the first 5 hours, media was then removed and cells incubated with 15% DMSO in Hanks' Buffered Saline Solution for 4 mins at 37°C. The solution was then removed, cells were rinsed with PBS and then incubated for a further 7h in phenol red free RPMI containing 10% human serum and 1% Penicillin-Streptomycin. Cells were then detached after 5 minutes incubation in 2mM EDTA in PBS$^{-/-}$, and expression of ALOX15 and EPHX2 was evaluated using flow cytometry. Cells were also suspended in PBS containing 0.1% human serum and incubated with *E. coli* as detailed above.

Mouse monocytes were isolated by adhesion of bone marrow cell suspension from WT and ALOX15-deficient mice to tissue culture dishes. After 45 min cell were detached and cell populations determined to be ~80-90% monocytes. Monocytes (2 × 10^6 cells/mL) were incubated in phenol red free RPMI containing 0.1% FBS and *E. coli* (1 × 10^8 CFU/mL) at 37°C for 45 min. Incubations were quenched using 2 volumes of ice-cold methanol containing deuterium labelled internal standards and lipid mediators identified and quantified using lipid mediator profiling.

Enzyme Incubations

Human recombinant (hr)-ALOX15 (0.2μM; Novus Biologicals) or (hr)-ALOX15B (0.2μM; Cayman Chemicals) was incubated with n-3 DPA (10 μM) in Tris buffer (pH = 8.0) at RT for 2 min. Products were quenched using excess acidified methanol (apparent pH ~3) containing deuterium labelled d_4-LTB_4 and extracted as detailed in the lipid mediator profiling section below. In select incubations hr-ALOX15 or hr-ALOX15B were incubated with EPHX2 (0.2μM; Cayman Chemicals) and n-3 DPA (10μM) in Tris buffer (pH = 8.0) at RT for 15 min. Incubations were quenched using ice-cold methanol and products extracted, identified and quantified as detailed below.

In select incubations EPHX2 (0.2μM) was incubated with 16*S*,17*S*-e$PD_{n\text{-}3\ DPA}$ (10nM) in Tris buffer (pH = 8.0) at RT for 10 min; incubations were then quenched, and products identified and quantified as detailed below.

Flow Cytometry

Flow cytometry was used to determine the phenotypic lineage of the monocyte-derived macrophages using fluorescently conjugated antibodies. Cells were fixed in 1% paraformaldehyde (4% paraformaldehyde in PBS$^{-/-}$) for 10 minutes at room temperature. Cells were incubated with the following antibodies in a 1:100 dilution for 30 minutes at 4°C in staining solution (1:1 PBS with 0.02 % BSA and FC block): APC/Cy7 anti-human CD14 (clone 63D3, Biolegend), PE-Cyanine7 anti-human CD32 (clone 6C4, eBiosciences), Alexa Fluor (AF) 405 anti-ICAM-1 (clone 1A29, Novus), PE/Cy5 anti-CD64 (clone 10.1, Abcam), AF 488 anti-human CD68 (clone Y1/82A, Biolegend), Brilliant Violet (BV) 650 anti-human CD80 (clone 2D10, Biolegend), PerCP/Cy5.5 anti-human CD206 (clone 15-2, Biolegend) and PE-CF594 anti-human CD163 (clone GHI/61, BD Biosciences). LSR Fortessa cell analyser (BD Biosciences) was used to perform multiparameter analysis, followed by analysis using FlowJo (Tree Star Inc., V10).

To determine the expression of $PD_{n\text{-}3\ DPA}$ biosynthetic enzymes, monocytes and macrophages were incubated with M-CSF and IL-4 or GM-CSF, IFNγ and LPS for 7 days as detailed above. At the indicated intervals cells were collected, permeabilized using eBiosciences Fixation/ Permeabilization Solution Kit following manufacturer's instructions, non-specific binding was quenched using non-specific IgG (16mg/mL) and cells were then incubated with rabbit anti-human 15-LOX type 1, mouse anti-human 15-LOX type 2, and mouse anti-human EH2 for 30 min at 4°C. To determine the expression of 15-LOX type 2 and EH2, cells were then incubated with AF 488 goat anti-mouse IgG for 30 min at 4°C and staining evaluated using LSR Fortessa cell analyser (BD Biosciences) was used to perform multiparameter data acquisition, followed by analysis using FlowJo (Tree Star Inc., V10).

In select experiments cells were collected from the peritoneum of WT and ALOX1- deficient mice by lavaging the peritoneum with PBS. Splenic cells were obtained following dissociation of spleens from WT and ALOX15-deficient mice using a 70 μM filter. The cells were counted and incubated with the following antibodies in a 1:100 dilution for 30 minutes at 4°C in staining solution (1:1 PBS with 0.02 % BSA and TrueStain FC Blocking IgG): PE-Cy5 anti-mouse/human CD 11b (Clone M1/70, Biolegend), BV 650 rat anti-mouse I-A/I-E (MHC II) (Clone M5/114.15.2, Biolegend), APC-Cy7 rat anti-mouse F4/80 (Clone BM8, Biolegend), BV785 hamster anti-mouse CD 11c (Clone N418, Biolegend) and PerCP-eFluor710 rat anti-mouse TIM-4 (Clone 54 (RMT4-54), eBiosciences). For intracellular staining, cells were permeabilized using eBiosciences Fixation/ Permeabilization Solution Kit following manufacturer's instructions for 20 min at RT and then with fluorescently labelled antibodies in a 1:50 dilution for 30 minutes at 4°C in staining solution

(1:10 Permeabilization buffer (10X) (eBiosciences) and PBS with 0.02 % BSA): AF 488 COX 2 (Clone (D5H5) XP ®, Cell Signaling Technologies), PE-Dazzle 594 rat anti-mouse IL-10 (Clone JES5-16E3, Biolegend), BV 421 mouse anti-mouse TGF-β1 (Clone TW7-16B4, Biolegend), PE sheep anti-mouse Arginase 1 (Cat # IC5868P, R&D) and AF 647 rabbit- anti-human iNOS (Clone 4E5, Novus). Non-specific binding was quenched using TruStain Fc-blocking IgG (anti-mouse CD16/32) and the staining was evaluated as above. For cells obtained from both *in vivo* efferocytosis and phagocytosis experiments non-sepcific binding was quenched as detailed above. Cells were then incubated in $PBS^{-/-}$ containing 0.02% BSA and either APC-Cy anti-mouse CD64 or PE anti-mouse CD64 (at 1:100 dilution) for 30 min at 4^oC and staining evaluated.

Macrophage Incubations

In Vitro *Efferocytosis*

Macrophages, prepared as described above, were seeded into 96 well plates at 5×10^4 cells per well in RPMI 1640 medium with 10 % human serum at 37°C. Apoptotic cells were prepared by incubating the human promyelocytic leukemic cell line HL60 at 70°C for 2h yielding ~60% Annexin V positive / propidium iodide negative cells and 35% Annexin V positive / propidium iodide positive cells using APC Annexin V Apoptotic Detection Kit with PI (Biolegend). These were then stained using PKH26 Red Fluorescent Cell Linker kit (Sigma) following manufacturer's instructions and efferocytosis was assessed as in (Dalli and Serhan, 2012). In brief, fluorescently labelled apoptotic cells (1:3, macrophages to apoptotic cells) were added to the macrophages and incubated at 37°C for 45 minutes. Cells were washed with PBS and extracellular fluorescence was quenched using trypan blue (1:15 in PBS). Fluorescence was then measured using a FLUOstar Omega microplate reader (BMG Labtech). In select experiments apoptotic cells were labelled using PKH67 and incubated with macrophages as detailed above. Macrophage efferocytosis was then evaluated using an ImageStream X MK2 and analysis was performed using IDEAS® (Image Data Exploration and Analysis Software, Version 6.0).

In Vivo *Efferocytosis*

Mice were administered either vehicle or $PD1_{n\text{-}3\ DPA}$ (10ng/mouse) via i.p. injection for 7 days. Apoptotic cells were prepared and labelled as detailed above using PKH67 dye. These ($6x10^6$ cells per mouse) were injected via intraperitoneal injection to WT and ALOX15-deficient mice. After 1h cells were collected by peritoneal lavages and incubated with an APC-Cy7 labelled anti-mouse CD64 antibody as detailed above and efferocytosis in CD64 positive macrophages was evaluated using Flow cytometry.

E. coli *Incubations*

Macrophages ($2x10^6$ cells) were incubated with *E. coli* ($1x10^7$ cells) for 30 minutes at 37°C. Incubations were quenched using two volumes of ice-cold methanol containing deuterium labelled internal standards (500 pg each of d_4-PGE_2, d_5-LXA_4, d_4-RvD2, d_4-LTB_4 and d_8-5S-HETE, Cayman Chemicals). Samples were then stored at -20°C prior to extraction and lipid mediator profiling.

In Vivo Phagocytosis

Mice were administered either vehicle or $PD1_{n\text{-}3\ DPA}$ (10ng/mouse) via i.p. injection for 7 days. *E. coli* were labelled using LIVE/DEAD BacLight Bacterial Viability dye following manufacturer's instructions. Fluorescently labelled bacteria were then injected *via* intraperitoneal injection to WT and ALOX15-deficient mice and peritoneal lavages were collected after 1h. Cells were stained using fluorescently labelled PE anti-mouse CD64 antibody and bacterial phagocytosis was evaluated using flow cytometry as detailed above.

Lipid Mediator Profiling

Proteins were allowed to precipitate by keeping samples at -20°C for 30 minutes. Deuterium-labelled standards, d_4-PGE_2, d_5-LXA_4, d_4-RvD2, d_4-LTB_4 and d_8-5S-HETE were used to aid in identification and quantification of lipid mediators (Dalli and Serhan, 2012). Following protein precipitation, samples were then extracted using an ExtraHera (Biotage) with ISOLUTE C18 columns (500 mg, 3 mL; Biotage). This involved the conditioning of the Solid-phase ISOLUTE C18 500 mg/3 mL columns with methanol for 60 seconds using 2.5 bar positive pressure. Samples, brought to 10 mL with pH 3.5 water, were loaded onto the columns for 90 seconds using 2.5 bar positive pressure. The acid in the C18 columns was neutralised by washing the columns with 2 mL pH 7 water for 45 seconds using 2.5 bar positive pressure, followed by the hexane wash to elute hydrophobic molecules. This was done four times with 3 mL hexane using 2.5 bar positive pressure for 60 seconds for each wash. Mediators were then eluted into collection tubes with the addition of 5 mL methyl formate at 1.5 bar positive pressure for 120 seconds. Products were brought to dryness using a gentle nitrogen stream and TurboVap LV (Biotage), these were then suspended in phase containing methanol and water, 1:1 (vol/vol).

An LC-MS-MS system, comprising of a Qtrap 5500 (AB Sciex) or Qtrap 6500 plus (AB Sciex), Shimadzu SIL-20AC autoinjector, LC-20AD binary pump (Shimadzu Corp.) and Agilent C18 Poroshell column (150 mm × 4.6 mm × 2.7 μm) was used to profile lipid mediators. The gradient was initiated at 20:80:0.01 (vol/vol/vol) methanol/water/acetic acid for 0.2 mins this was ramped to 50:50:0.01 (vol/vol/vol) over 12 seconds, maintained for 2 minutes, then ramped to 80:20:0.01 (vol/vol/vol) over 9 minutes, and maintained for 3.5 minutes. The ratio was then ramped to 98:2:0.01 (vol/vol/vol) for 5.5 minutes. The flow rate was kept at 0.5 mL/minute throughout.

Mediator concentrations were determined using multiple reaction monitoring (MRM) using signature parent ion (Q1) and characteristic daughter ion (Q3) pairs. A minimum of six diagnostic ions were used to confirm their identities, using published criteria (Dalli and Serhan, 2012). The peak area of the MRM transition and linear calibration curves with an r^2 value of 0.98 to 0.99 were used to quantify each of the molecules. The detection limit was ~0.1 pg.

Total Organic Synthesis of 16*S*,17*S*-ePD$_{n\text{-}3\ DPA}$

Unless stated otherwise, all commercially available reagents and solvents were used in the form they were supplied without any further purification. The stated yields are based on isolated material. All reactions were performed under an argon atmosphere using Schlenk techniques. Reaction flasks were covered with aluminium foil during reactions and storage to minimize exposure to sunlight. Thin layer chromatography was performed on silica gel 60 F254 aluminium-backed plates fabricated by Merck. Flash column chromatography was performed on silica gel 60 (40-63 μm) produced by Merck. NMR spectra were recorded on a Bruker AVI600, Bruker AVII400 or a Bruker DPX300 spectrometer at 600 MHz, 400 MHz or 300 MHz respectively for ^{1}H NMR and at 150 MHz, 100 MHz or 75 MHz respectively for ^{13}C NMR. Coupling constants (*J*) are reported in hertz and chemical shifts are reported in parts per million (δ) relative to the central residual protium solvent resonance in ^{1}H NMR ($CDCl_3$ = δ 7.26, DMSO-d_6 = δ 2.50, benzene-d_6 = δ 7.16 and MeOD-d_4 = δ 3.31 ppm) and the central carbon solvent resonance in ^{13}C NMR (CDCl3 = δ 77.00, DMSO-d_6 = δ 39.43, benzene-d_6 = δ 128.06 and MeOD-d_4 = δ 49.00 ppm). Mass spectra were recorded at 70 eV on Micromass Prospec Q or Micromas QTOF 2W spectrometer using EI, ES or CI as the methods of ionization. High resolution mass spectra were recorded on Micromass Prospec Q or Micromas QTOF 2Wspectrometer using EI or ES as the methods of ionization. Optical rotations were measured using a 0.7 mL cell with a 1.0 dm path length on an Anton Paar MCP 100 polarimeter. HPLC analyses were performed on an Agilent Technologies 1200 Series instrument with diode array detector set at 254 nm and equipped with a C18 stationary phase (Eclipse XDB-C18 5 μm 4.6 × 150 mm), applying the conditions stated. GLC analyses were performed on an Agilent 7820A with a FID detector, HP-5 capillary column, with helium as the carrier gas and by applying the conditions stated.

Synthesis of methyl (7Z,10Z,12E,14E)-15-((2S,3S)-3-((Z)-pent-2-en-1-yl)oxiran-2-yl)pentadeca-7,10,12,14-tetraenoate (methyl ePD1$_{n\text{-}3\ DPA}$)

The epoxy aldehyde (2*E*,4*E*)-5-((2*S*,3*S*)-3-((*Z*)-pent-2 en-1-yl)oxiran-2-yl)penta-2,4-dienal was prepared from commercially available 2-(triphenyl-λ^5-phosphanylidene)acetaldehyde and (2*R*,3*S*)-3-((*Z*)-pent-2-en-1-yl)oxirane-2-carbaldehyde as described (Aursnes et al., 2015). The Wittig-salt methyl (*Z*)-10-(iodotriphenyl-λ^5-phosphanyl)dec-7-enoate was made according to literature protocols (Primdahl et al., 2017)

Z-Selective Wittig Reaction

This reaction was performed as earlier reported (Aursnes et al., 2014). In brief, 1.3 equiv. of the Wittig salt methyl (*Z*)-10-(iodotriphenyl-λ^5-phosphanyl)dec-7-enoate was dissolved in THF and then HMPA was added. This solution was cooled to −78°C. Then NaHMDS (0.60 M in toluene, 1.3 equiv.) was added drop-wise. The reaction mixture was stirred for 30 min at this temperature. The epoxy aldehyde (2*E*,4*E*)-5-((2*S*,3*S*)-3-((*Z*)-pent-2 en-1-yl)oxiran-2-yl)penta-2,4-dienal (1.0 equiv.) was azeotroped twice with 2-methyltetrahydrofuran, then dissolved in THF, and cooled to −78°C. This solution was added dropwise to the reaction flask via cannula and the resulting reaction mixture was stirred for 1 h at −78°C. Then the temperature of the reaction mixture was quickly warmed to −20°C and kept at this temperature for a few minutes. The reaction mixture was then quenched by the addition of an equal amount of phosphate buffer (0.2 M, pH = 7) as the volume of the reaction mixture. The phases were separated, and the aqueous phase was extracted with Et_2O. The combined organic layers were dried with Na_2SO_4, then filtrated, and concentrated in vacuo. The resulting oil was purified by silica gel chromatography that had been deactivated by a solution containing Et_3N (3%), Et_2O (20%) in heptane. The product was eluted with Et_2O (25%) in heptane to provide the titled epoxy methyl ester as a colorless oil. Physical and spectral data: $[\alpha]^{20}_{D}$ = −38 (c = 0.20, $CHCl_3$); UV (hexane) λ_{max} 270, 281, 292; ^{1}H NMR (600 MHz, benzene-d_6) δ 6.54 (dd, *J* = 14.7, 11.4 Hz, ^{1}H), 6.39 (dd, *J* = 15.3, 10.8 Hz, ^{1}H), 6.11 (dd, *J* = 14.8, 10.9 Hz, ^{1}H), 6.04 (t, *J* = 11.2 Hz, ^{1}H), 5.51 – 5.31 (m, ^{6}H), 3.36 (s, ^{3}H), 3.06 (dd, *J* = 7.8, 1.8 Hz, ^{1}H), 2.91 (t, *J* = 7.1 Hz, ^{2}H), 2.71 (td, *J* = 5.2, 2.0 Hz, ^{1}H), 2.24 (dq, *J* = 13.8, 6.4 Hz, ^{1}H), 2.20 – 2.14 (m, ^{1}H), 2.09 (t, *J* = 7.4 Hz, ^{2}H), 1.94 (dq, *J* = 23.1, 8.4, 7.5 Hz, ^{4}H), 1.53 (p, *J* = 7.4 Hz, ^{2}H), 1.25 – 1.13 (m, 4H), 0.88 (t, *J* = 7.5 Hz, ^{3}H). ^{13}C NMR (151 MHz, C_6D_6) δ 173.3, 134.7, 134.1, 132.3, 131.4, 131.4, 130.8, 129.0, 128.9, 127.6, 123.2, 60.3, 57.7, 51.0, 34.1, 30.1, 29.6, 29.0, 27.4, 26.7, 25.2, 21.0, 14.4; HRMS (TOF ES$^+$): Exact mass calculated for $C_{23}H_{34}O_3Na$ $[M+Na]^+$: 381.2400, found 381.2400. TLC (heptane/Et_2O, 74:26 CAM stain): R_f = 0.21.

(7Z,10Z,12E,14E)-15-((2S,3S)-3-((Z)-pent-2-en-1-yl)oxiran-2-yl)pentadeca-7,10,12,14-tetraenoic acid (ePD$_{n\text{-}3\ DPA}$). Methyl (7Z,10Z,12E,14E)-15-((2S,3S)-3-((Z)-pent-2-en-1-yl)oxiran-2-yl)pentadeca-7,10,12,14-tetraenoate (100 μg in hexane/Et_2O) was dried under a gentle stream of nitrogen and then dissolved in 500 μL of THF. For additional details on the preparation of this compound, see (Primdahl et al., 2017). This THF solution was cooled to -78°C using a dry ice/isopropanol cooling bath. Then 100 μL of aqueous 1.0 M LiOH solution was slowly added via a Hamilton syringe at -78°C. Additional 100 μL of H_2O was added and the vial was covered with aluminium foil and left stirring for 10 h. The solution above the precipitated lithium salt was gently removed by using a syringe. Next, the THF-solution was dried under a gentle stream of nitrogen before quantification using UV (hexane) λ_{max} 271 (log ε 3.58), 280 (log ε 4.08), 298 (log ε 2.69) nm. After quantification and removal of hexane, the acid was suspended in 50 μL PBS$^{+/+}$ pH = 7.45. The PBS solution was kept on solid dry ice in a closed container prior to use, and was used for incubation biological experiments. The structure of the chemical labile free acid was determined indirectly by using UV and LC/MS-MS experiments.

QUANTIFICATION AND STATISTICAL ANALYSIS

Results are represented as mean ± SEM. Differences between groups were assessed using one-sample t test (normalized data), Student's t test (2 groups), 1-way ANOVA (multiple groups) followed by post hoc Dunnett's test using GraphPad Prism 6 software. Investigators were not blinded to group allocation or outcome assessment. The criterion for statistical significance was $P \leq 0.05$.

Sample sizes for each experiment were determined on the variability observed in prior experiments (Dalli and Serhan, 2016) and preliminary experiments. Partial least squares-discrimination analysis (PLS-DA) and principal component analysis (PCA) (Janes and Yaffe, 2006) were performed using SIMCA 14.1 software (Umetrics, Umea, Sweden) following mean centering and unit variance scaling of LM levels. PLS-DA is based on a linear multivariate model that identifies variables that contribute to class separation of observations on the basis of their variables (LM levels). During classification, observations were projected onto their respective class model. The score plot illustrates the systematic clusters among the observations (closer plots presenting higher similarity in the data matrix). Loading plot interpretation identified the variables with the best discriminatory power (Variable Importance in Projection greater than 1) that were associated with the distinct intervals and contributed to the tight clusters observed in the Score plot.

DATA AND SOFTWARE AVAILABILITY

Data are available upon request to the Lead contact.

Cell Chemical Biology, Volume *25*

Supplemental Information

$PD_{n\text{-}3\ DPA}$ Pathway Regulates Human Monocyte Differentiation and Macrophage Function

Kimberly Pistorius, Patricia R. Souza, Roberta De Matteis, Shani Austin-Williams, Karoline G. Primdahl, Anders Vik, Francesca Mazzacuva, Romain A. Colas, Raquel M. Marques, Trond V. Hansen, and Jesmond Dalli

Figure S1

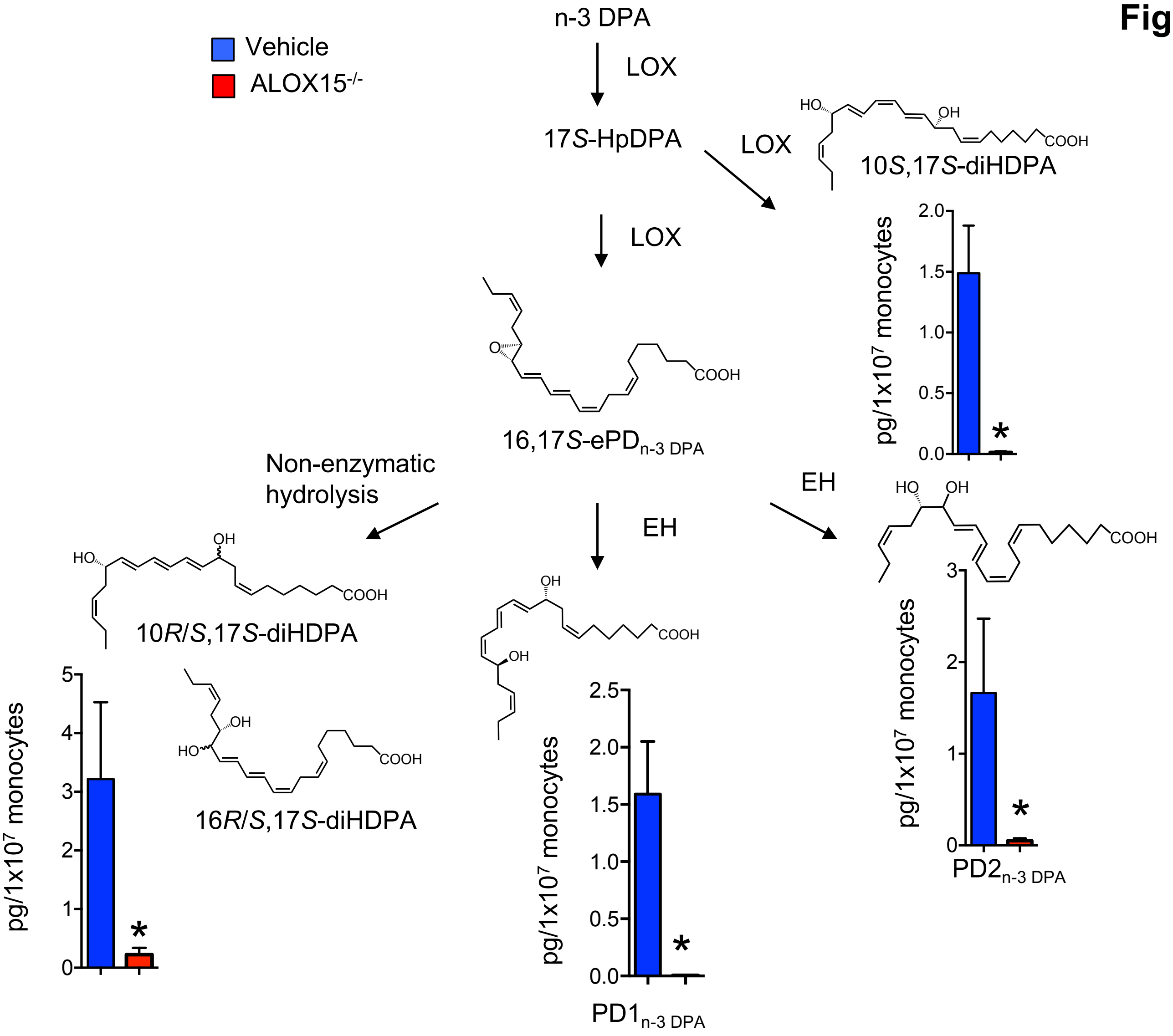

Vehicle
ALOX15-/-
n-3 DPA
LOX
17S-HpDPA
LOX
10S,17S-diHDPA
LOX
16,17S-ePDn-3 DPA
Non-enzymatic hydrolysis
EH
EH
10R/S,17S-diHDPA
16R/S,17S-diHDPA
PD1n-3 DPA
PD2n-3 DPA
pg/1x10^7 monocytes
COOH
HO
OH
O
*

Figure S2

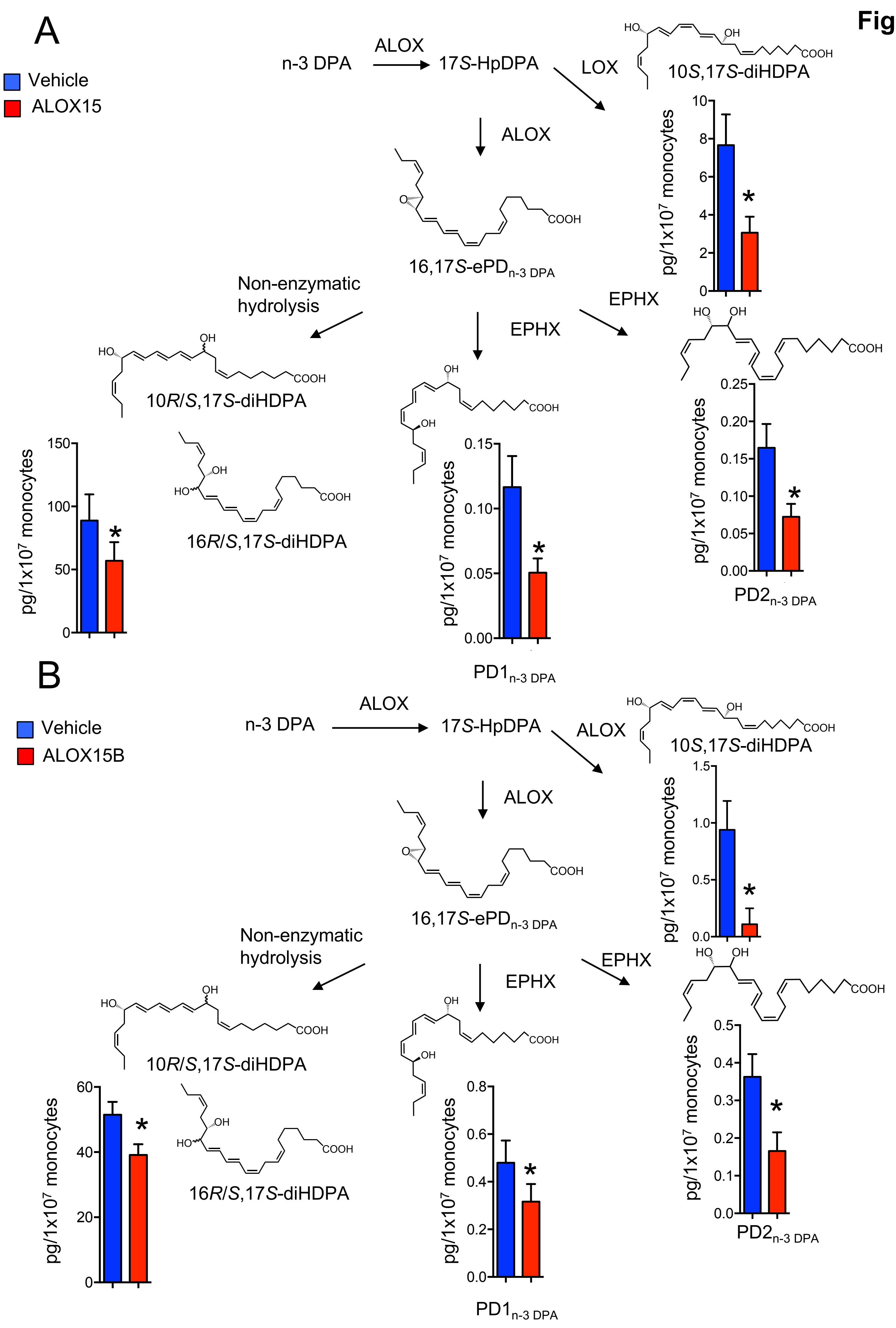
A
Vehicle
ALOX15
n-3 DPA
ALOX
17S-HpDPA
LOX
10S,17S-diHDPA
ALOX
16,17S-ePD n-3 DPA
Non-enzymatic hydrolysis
EPHX
EPHX
10R/S,17S-diHDPA
16R/S,17S-diHDPA
PD1 n-3 DPA
PD2 n-3 DPA
pg/1x10^7 monocytes
B
Vehicle
ALOX15B
n-3 DPA
ALOX
17S-HpDPA
ALOX
10S,17S-diHDPA
ALOX
16,17S-ePD n-3 DPA
Non-enzymatic hydrolysis
EPHX
EPHX
10R/S,17S-diHDPA
16R/S,17S-diHDPA
PD1 n-3 DPA
PD2 n-3 DPA
pg/1x10^7 monocytes

Figure S3

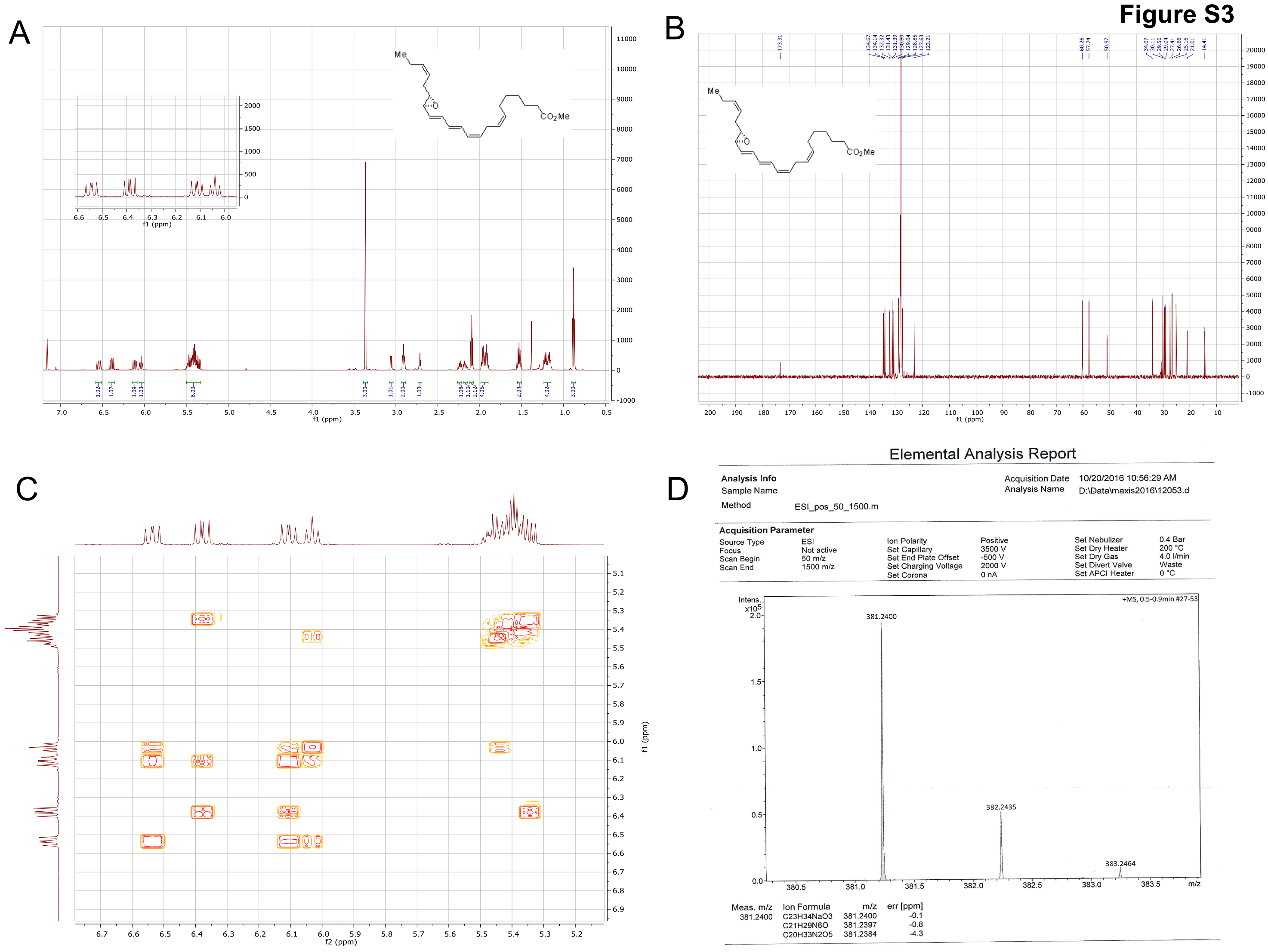
A
B
C
D
Me
CO_2Me
f1 (ppm)
f2 (ppm)
Elemental Analysis Report
Analysis Info
Sample Name
Method ESI_pos_50_1500.m
Acquisition Date 10/20/2016 10:56:29 AM
Analysis Name D:\Data\maxis2016\12053.d
Acquisition Parameter
Source Type ESI
Focus Not active
Scan Begin 50 m/z
Scan End 1500 m/z
Ion Polarity Positive
Set Capillary 3500 V
Set End Plate Offset -500 V
Set Charging Voltage 2000 V
Set Corona 0 nA
Set Nebulizer 0.4 Bar
Set Dry Heater 200 °C
Set Dry Gas 4.0 l/min
Set Divert Valve Waste
Set APCI Heater 0 °C
+MS, 0.5-0.9min #27-53
381.2400
382.2435
383.2464
Meas. m/z 381.2400
Ion Formula C23H34NaO3 381.2400 -0.1
C21H29N6O 381.2397 -0.8
C20H33N2O5 381.2384 -4.3

Figure S4

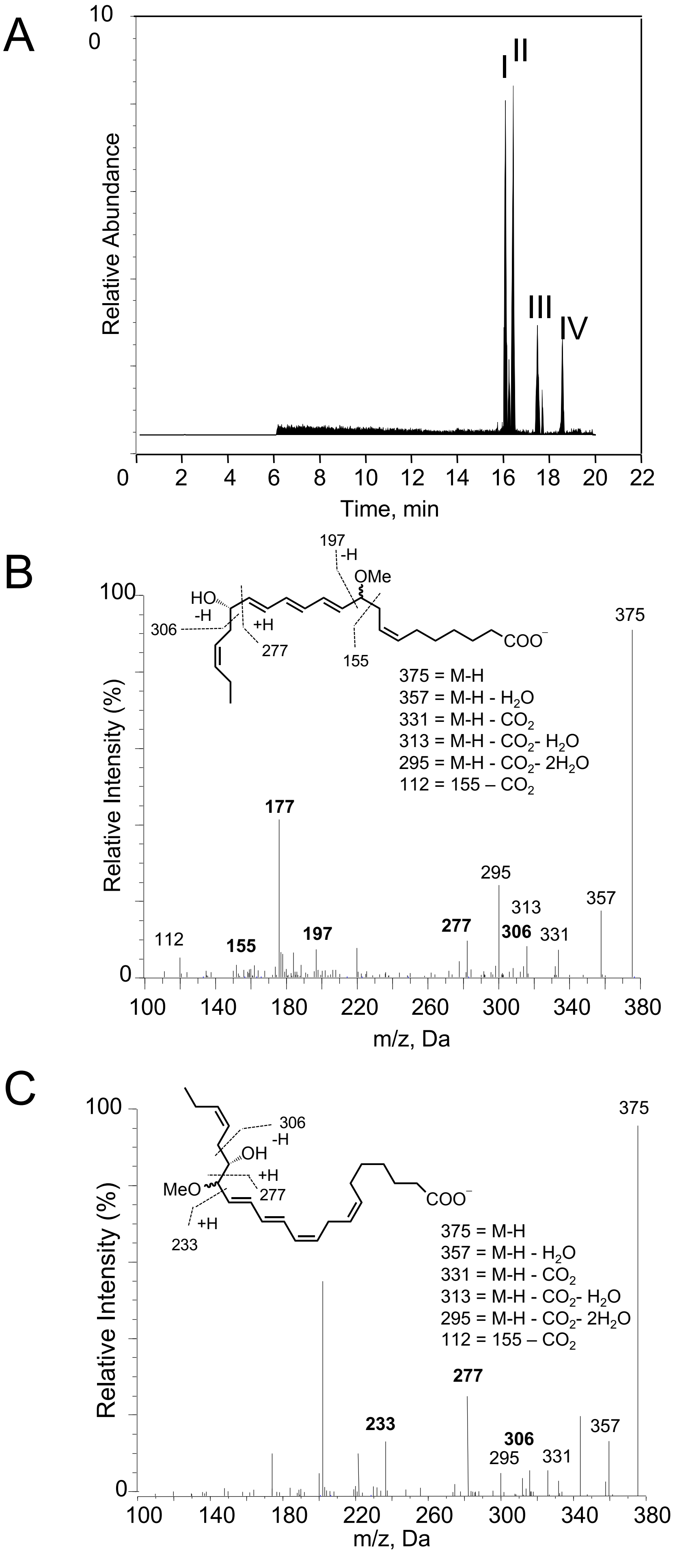

A
Relative Abundance
100
I
II
III
IV
0 2 4 6 8 10 12 14 16 18 20 22
Time, min
B
197
-H
OMe
HO
-H
306
+H
277
155
COO⁻
375 = M-H
357 = M-H - H2O
331 = M-H - CO2
313 = M-H - CO2- H2O
295 = M-H - CO2- 2H2O
112 = 155 – CO2
Relative Intensity (%)
100
0
375
177
295
313
357
112
155
197
277
306
331
100 140 180 220 260 300 340 380
m/z, Da
C
306
-H
OH
+H
277
MeO
+H
233
COO⁻
375 = M-H
357 = M-H - H2O
331 = M-H - CO2
313 = M-H - CO2- H2O
295 = M-H - CO2- 2H2O
112 = 155 – CO2
Relative Intensity (%)
100
0
375
277
233
306
357
295
331
100 140 180 220 260 300 340 380
m/z, Da

Figure S5

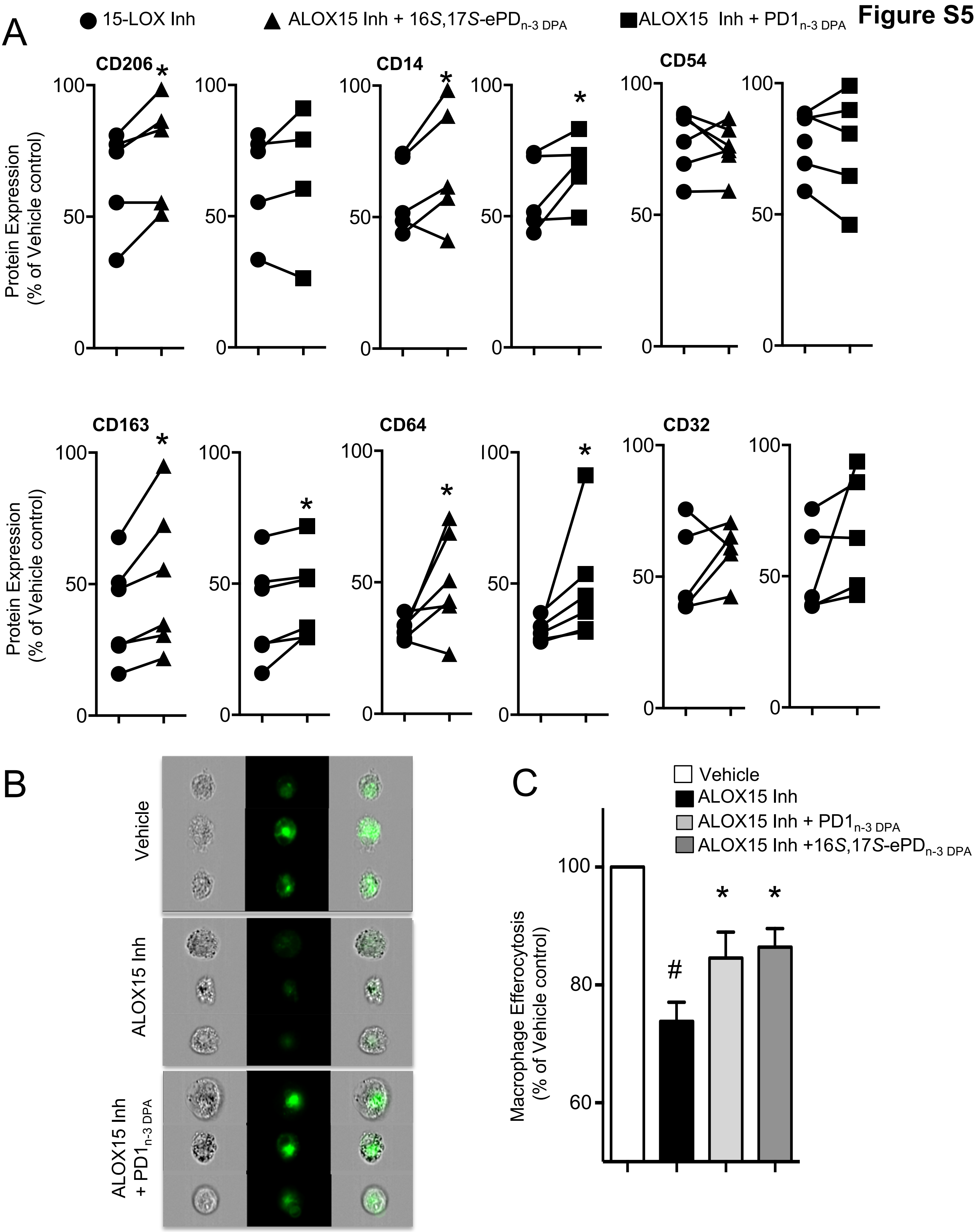

A
15-LOX Inh
ALOX15 Inh + 16S,17S-ePD n-3 DPA
ALOX15 Inh + PD1 n-3 DPA
CD206
CD14
CD54
CD163
CD64
CD32
Protein Expression (% of Vehicle control)
0
50
100
*
B
Vehicle
ALOX15 Inh
ALOX15 Inh + PD1 n-3 DPA
C
Vehicle
ALOX15 Inh
ALOX15 Inh + PD1 n-3 DPA
ALOX15 Inh +16S,17S-ePD n-3 DPA
Macrophage Efferocytosis (% of Vehicle control)
60
80
100
#
*

Supplemental Figures

Figure S1: Decreased $PD_{n\text{-}3\ DPA}$ in ALOX15 deficient mice. Related to Figure 1 and Table S1.
Bone marrow monocytes were isolated from WT or ALOX15 deficient mice. These ($2x10^6$ cells/ml) were then incubated with *E. coli* ($1x10^8$ CFU/mL) for 30 min (37°C; $PBS^{+/+}$). Incubations were quenched with ice-cold methanol and products identified and quantified using lipid mediator profiling. Results are mean ± SEM. n = 4 mice per group. * $P<0.05$.

Figure S2: Knockdown of both ALOX15 and ALOX15B reduces $PD_{n\text{-}3\ DPA}$ biosynthesis in human monocytes. Related to Figure 1.
Human monocytes were transfected with shRNA to (A) ALOX15, (B) ALOX15B or CT shRNA (see methods for details). The cells ($1x10^6$ cells/mL) were incubated with *E. coli* ($5x10^7$ CFU/mL) for 45 min (37°C) and $PD_{n\text{-}3\ DPA}$ levels were ascertained using lipid mediator profiling. Results are mean ± SEM. n = 5 donors.

Figure S3: Characterizing the synthetic 16*S*,17*S*-$ePD_{n\text{-}3\ DPA}$. Related to Figure 3. (A) ^{1}H-NMR spectrum of methyl (7*Z*,10*Z*,12*E*,14*E*)-15-((2*S*,3*S*)-3-((*Z*)-pent-2-en-1-yl)oxiran-2-yl)pentadeca-7,10,12,14-tetraenoate (methyl $ePD1_{n\text{-}3\ DPA}$). (B) ^{13}C-NMR spectrum of methyl (7*Z*,10*Z*,12*E*,14*E*)-15-((2*S*,3*S*)-3-((*Z*)-pent-2-en-1-yl)oxiran-2-yl)pentadeca-7,10,12,14-tetraenoate (methyl $ePD1_{n\text{-}3\ DPA}$). (C) HSQC spectrum of methyl (7*Z*,10*Z*,12*E*,14*E*)-15-((2*S*,3*S*)-3-((*Z*)-pent-2-en-1-yl)oxiran-2-yl)pentadeca-7,10,12,14-tetraenoate (methyl $ePD1_{n\text{-}3\ DPA}$). (D) HRMS of methyl (7*Z*,10*Z*,12*E*,14*E*)-15-((2*S*,3*S*)-3-((*Z*)-pent-2-en-1-yl)oxiran-2-yl)pentadeca-7,10,12,14-tetraenoate (methyl $ePD1_{n\text{-}3\ DPA}$).

Figure S4: Acid alcohol trapping product profile for synthetic 16*S*,17*S*-$ePD_{n\text{-}3\ DPA}$. Related to Figure 2.
The epoxide was incubated in acidified methanol and products were profiled using LC/MS-MS. (A) MRM chromatogram for ion pairs *m/z* 375>277. (B-C) MS-MS spectra employed in the identification of (B) 10-methoxy,17*S*-hydroxy-7*Z*,11*E*,13*E*,15*E*,19Z-docosapentaenoic acid and (C) 16-methoxy,17S-hydroxy-7Z,10Z,12E,14E,19Z-docosapentaenoic acid in monocyte incubations.

Figure S5: 16*S*,17*S*-$ePD_{n\text{-}3\ DPA}$ and $PD1_{n\text{-}3\ DPA}$ rectify monocyte differentiation following ALOX15 inhibition restoring macrophage responses. Related to Figure 7.
Human monocytes were incubated with M-CSF (20ng/mL) and 15-LOX inhibitor (10μM), ALOX15 inhibitor + 16*S*, 17*S*-$ePD_{n\text{-}3\ DPA}$ (1nM), ALOX15 inhibitor + $PD1_{n\text{-}3\ DPA}$ (1nM), or vehicle (37°C, 5% CO_2 in RPMI supplemented with human serum). On day 7 (A) cells were collected and expression of lineage markers was determined using fluorescently labelled antibodies and flow cytometry. (B-C) cells were incubated with fluorescently labelled apoptotic cells for 45 min (37°C) and phagocytosis was evaluated using (B) ImageStream (C) fluorescent plate reader . * $P < 0.05$ vs ALOX15 inhibitor incubations; # $P < 0.05$ versus vehicle incubations.

Supplemental Tables

Table S1: ALOX15 inhibitor shifts human monocyte-derived macrophage lipid mediator profiles Related to Figure 1.

DHA bioactive metabolome	Q1	Q3	Vehicle	ALOX15 inhibitor
RvD1	375	141	26.8 ± 4.6	13.3 ± 3.3 *
RvD2	375	141	127.6 ± 42.8	62.9 ± 42.5 *
RvD3	375	147	19.3 ± 7.6	10.4 ± 5.2
RvD4	375	101	172.6 ± 33.9	127.7 ± 42.7
RvD5	359	199	104.1 ± 37.4	65.6 ± 31.9 *
RvD6	359	101	639.4 ± 233.8	532.5 ± 187.5 *
17R-RvD1	375	141	47.0 ± 18.8	19.0 ± 5.7
17R-RvD3	375	147	12.5 ± 4.2	8.3 ± 4.3
PD1	359	153	24.4 ± 6.2	16.6 ± 5.7 *
10S,17S-diHDHA	359	153	309.0 ± 114.3	220.8 ± 92.3 *
17R-PD1	359	153	18.7 ± 10.7	17.0 ± 12.3
22-OH-PD1	375	153	7.4 ± 3.8	4.8 ± 3.1 *
MaR1	359	221	64.2 ± 56.6	22.1 ± 10.5
MaR2	359	191	184.0 ± 98.9	161.2 ± 88.0
7S,14S-diHDHA	359	221	259.9 ± 87.3	427.0 ± 143.6
22-OH-MaR1	375	221	196.1 ± 79.5	200.1 ± 74.6
4S,14S-diHDHA	359	101	69.7 ± 61.7	20.5 ± 9.9
14-oxo-MaR1	357	248	16.3 ± 7.4	9.5 ± 5.4
**n-3 DPA bioactive metabolome**				
RvT1	377	239	39.9 ± 21.2	26.7 ± 12.3
RvT2	377	197	34.6 ± 21.6	25.8 ± 16.7
RvT3	377	197	22.1 ± 12.8	10.0 ± 3.9
RvT4	361	211	36.0 ± 11.2	62.7 ± 34.6
$RvD1_{n\text{-}3\ DPA}$	377	143	39.8 ± 16.5	28.0 ± 15.0 *
$RvD2_{n\text{-}3\ DPA}$	377	143	21.2 ± 8.5	12.9 ± 3.1
$RvD5_{n\text{-}3\ DPA}$	361	199	125.2 ± 32.7	76.6 ± 16.1 *
$PD1_{n\text{-}3\ DPA}$	361	183	32.5 ± 9.1	11.0 ± 1.2 *
$PD2_{n\text{-}3\ DPA}$	361	263	27.5 ± 10.0	13.4 ± 8.2 *
10S, 17S-diHDPA	361	183	33.9 ± 7.6	19.5 ± 3.6 *
Δ15trans-$PD1_{n\text{-}3\ DPA}$	361	183	61.0 ± 25.9	52.2 ± 23.4 *
10epi-Δ15trans-$PD1_{n\text{-}3\ DPA}$	361	183	75.3 ± 19.4	55.0 ± 20.8 *
$MaR1_{n\text{-}3\ DPA}$	361	249	13.8 ± 7.3	18.2 ± 11.4
7S, 14S-diHDPA	361	249	77.9 ± 28.2	47.6 ± 14.2
**EPA bioactive metabolome**				
RvE1	349	195	18.5 ± 11.9	18.6 ± 12.7
RvE2	333	199	269.5 ± 102.2	246.0 ± 112.1
RvE3	333	201	93.8 ± 33.6	90.7 ± 39.9
**AA bioactive metabolome**				
LXA_4	351	217	514.2 ± 307.5	422.7 ± 239.5
LXB_4	351	221	602.9 ± 280.3	492.4 ± 228.9 *
5S,15S-diHETE	335	235	5259.1 ± 1992.8	3585.6 ± 1638.2 *
15R-LXA_4	351	217	386.2 ± 172.4	308.7 ± 146.9
15R-LXB_4	351	221	1848.8 ± 855.8	1283.9 ± 516.3
LTB_4	335	195	175.8 ± 170.4	60.1 ± 58.7
5S,12S-diHETE	335	195	1145.4 ± 596.4	1073.1 ± 586.4
20-OH-LTB_4	351	195	1.9 ± 0.6	1.4 ± 0.5
PGD_2	351	189	2191.3 ± 326.6	1277.4 ± 251.7 *
PGE_2	351	189	1983.8 ± 901.7	1151.9 ± 507.9
$PGF_{2\alpha}$	353	193	466.7 ± 196.9	230.1 ± 45.7
TxB_2	369	169	63661.3 ± 43046.4	19094.8 ± 14290.9

Human monocytes were incubated with M-CSF (20ng/ml) and either a ALOX15 inhibitor or vehicle (37°C, 5% CO_2). On day 7 incubations were quenched with ice-cold methanol and lipid mediators identified using LC/MS-MS based profiling. Results are mean $\pm$ SEM and expressed in pg/1x10^7 cells. Q1, M-H (parent ion); and Q3, diagnostic ion in the MS-MS (daughter ion). * $p<0.05$ using Mann Whitney test.

Table S2: Altered lipid mediator profiles in monocytes from ALOX15 deficient mice. Related to Figure 1 and Supplemental Figure 1.

DHA bioactive metabolome	Q1	Q3	WT	ALOX15$^{-/-}$
RvD1	375	141	1.63 ± 0.78	0.19 ± 0.20
RvD2	375	141	2.00 ± 1.37	0.20 ± 0.17
RvD3	375	147	0.17 ± 0.08	0.01 ± 0.02
RvD4	375	101	1.96 ± 1.31	0.14 ± 0.07
RvD5	359	199	23.03 ± 11.87	2.99 ± 2.14
RvD6	359	101	0.60 ± 0.34	0.00 ± 0.00
17R-RvD1	375	141	0.83 ± 0.30	0.08 ± 0.08 *
17R-RvD3	375	147	0.66 ± 0.34	0.10 ± 0.06
PD1	359	153	2.08 ± 1.15	0.13 ± 0.07
10S,17S-diHDHA	359	153	31.28 ± 17.71	0.30 ± 0.19
17R-PD1	359	153	1.69 ± 1.17	0.02 ± 0.01
22-OH-PD1	375	153	0.16 ± 0.11	0.05 ± 0.04
MaR1	359	221	17.48 ± 4.89	0.57 ± 0.62 *
MaR2	359	191	1.55 ± 1.17	0.07 ± 0.07
7S,14S-diHDHA	359	221	8.52 ± 4.03	0.14 ± 0.12 *
22-OH-MaR1	375	221	2.70 ± 2.40	0.32 ± 0.32
4S,14S-diHDHA	359	101	3.22 ± 2.30	0.15 ± 0.14
14-oxo-MaR1	357	248	1.20 ± 0.95	0.11 ± 0.10
**n-3 DPA bioactive metabolome**				
RvT1	377	239	15.45 ± 4.39	1.40 ± 1.46 *
RvT2	377	197	9.08 ± 4.20	0.97 ± 0.43 *
RvT3	377	197	2.93 ± 1.67	0.11 ± 0.07
RvT4	361	211	34.31 ± 19.67	0.91 ± 1.03
$RvD1_{n\text{-}3\ DPA}$	377	143	2.48 ± 2.06	0.33 ± 0.20
$RvD2_{n\text{-}3\ DPA}$	377	143	0.97 ± 0.52	0.15 ± 0.09
$RvD5_{n\text{-}3\ DPA}$	361	199	3.67 ± 0.13	1.08 ± 0.23 *
$PD1_{n\text{-}3\ DPA}$	361	183	1.59 ± 0.53	0.00 ± 0.00 *
$PD2_{n\text{-}3\ DPA}$	361	263	1.86 ± 0.86	0.05 ± 0.03 *
10S, 17S-diHDPA	361	183	1.49 ± 0.45	0.01 ± 0.01 *
Δ15trans-$PD1_{n\text{-}3\ DPA}$	361	183	1.93 ± 0.99	0.08 ± 0.04 *
10epi-Δ15trans-$PD1_{n\text{-}3\ DPA}$	361	183	1.49 ± 0.45	0.14 ± 0.10 *
$MaR1_{n\text{-}3\ DPA}$	361	249	0.70 ± 0.48	0.08 ± 0.08
7S, 14S-diHDPA	361	249	8.85 ± 5.22	0.57 ± 0.43
**EPA bioactive metabolome**				
RvE1	349	195	2.36 ± 1.43	0.28 ± 0.24
RvE2	333	199	1.39 ± 0.95	0.25 ± 0.15
RvE3	333	201	3.11 ± 1.88	0.42 ± 0.42
**AA bioactive metabolome**				
LXA_4	351	217	1.66 ± 0.69	0.03 ± 0.02 *
LXB_4	351	221	12.41 ± 4.13	4.86 ± 3.35
5S,15S-diHETE	335	235	84.99 ± 58.58	6.92 ± 4.78
15R-LXA_4	351	217	2.02 ± 0.83	6.03 ± 4.01
15R-LXB_4	351	221	1.92 ± 1.12	0.28 ± 0.14
LTB_4	335	195	348.45 ± 98.93	97.71 ± 83.25 *
5S,12S-diHETE	335	195	0.00 ± 0.00	0.00 ± 0.00
20-OH-LTB_4	351	195	2.73 ± 1.81	0.02 ± 0.02
PGD_2	351	189	1256.77 ± 411.18	129.81 ± 104.81 *
PGE_2	351	189	397.88 ± 215.48	32.80 ± 26.30
$PGF_{2\alpha}$	353	193	730.81 ± 330.29	71.06 ± 65.35 *
TxB_2	369	169	2374.02 ± 784.55	131.47 ± 112.91 *

Bone marrow monocytes ($2x10^6$ cells/mL) were incubated with *E. coli* ($2x10^8$ cells/mL) for 45 minutes at 37°C. Incubations were quenched with ice-cold methanol and lipid mediators identified using LC/MS-MS based profiling. Results are mean ± SEM and expressed in pg/$1x10^7$ cells. Q1, M-H (parent ion); and Q3, diagnostic ion in the MS-MS (daughter ion). * $p<0.05$ using Mann Whitney test.

Table S3: Tissue resident macrophages from ALOX15 mice display and altered expression of lineage markers that is rescued by $PD1_{n-3\ DPA}$ administration. Related to Figure 1 and Figure 7.

Large Peritoneal Macrophages			
**Lineage Marker**	**WT**	**12/15-LOX$^{-/-}$**	**12/15-LOX$^{-/-}$ + $PD1_{n-3\ DPA}$**
TIM 4	11479.5 ± 1285.4	7487.1 ± 1108.7 *	10738.4 ± 1767.6
CD64	5112.7 ± 460.6	5299.6 ± 478.1	5267.6 ± 802.9
TGFβ	924.9 ± 112.0	647.1 ± 124.2	1106.9 ± 162.8 #
MHCII	10338.4 ± 772.9	9914.0 ± 1200.7	9471.4 ± 1791.3
Arg-1	1192.0 ± 254.3	2004.1 ± 223.5 *	1398.8 ± 333.3
IL-10	621.4 ± 86.9	911.1 ± 46.0 **	787.4 ± 113.8
CD11b	7844.2 ± 856.0	11380.1 ± 737.9 **	9023.9 ± 1028.7 #

Small Peritoneal Macrophages			
**Lineage Marker**	**WT**	**ALOX15$^{-/-}$**	**ALOX15$^{-/-}$ + $PD1_{n-3\ DPA}$**
TIM-4	1741.6 ± 160.8	1129.3 ± 175.9 *	1680.4 ± 313.8
CD64	7908.3 ± 569.2	10092.6 ± 698.8 *	8196.0 ± 984.2
TGFβ	117.2 ± 7.6	181.5 ± 15.4 **	140.1 ± 12.7 #
MHCII	5972.0 ± 250.4	4923.7 ± 335.9 *	5943.4 ± 767.6
Arg-1	4284.9 ± 354.0	3356.3 ± 219.6 *	4160.4 ± 544.7
IL-10	365.1 ± 25.7	452.6 ± 41.4	357.9 ± 14.2 #
CD11b	296952.6 ± 111533.7	125432.1 ± 30807.4	490012.7 ± 204253.1 #

Splenic Macrophages			
**Lineage Marker**	**WT**	**ALOX15$^{-/-}$**	**ALOX15$^{-/-}$ + $PD1_{n-3\ DPA}$**
PTGS2	701.0 ± 22.2	894.7 ± 4.4 **	792.3 ± 14.4 ##
iNOS	3822.8 ± 273.1	4589.0 ± 184.4	4066.7 ± 290.0
CD64	7528.5 ± 468.8	9644.3 ± 292.0 *	8484.7 ± 315.6
TGFβ	72.2 ± 9.3	118.7 ± 7.2 *	96.2 ± 9.9
MHCII	11416.3 ± 461.6	10378.7 ± 369.3	10847.0 ± 247.2
CD11c	347.8 ± 6.0	501.0 ± 33.6 *	405.7 ± 18.8
Arg-1	70.6 ± 7.6	170.0 ± 10.6 **	129.3 ± 4.1 #
IL-10	123.0 ± 2.0	154.7 ± 2.2 **	136.7 ± 1.7 #
CD11b	2129.0 ± 172.6	4029.7 ± 174.9 **	2476.7 ± 92.3 #

Tissue resident macrophages were collected from WT mice given vehicle (PBS) or ALOX$^{-/-}$ mice given either vehicle of 10ng $PD1_{n-3\ DPA}$ daily for 7 days. The expression of lineage markers was determined using flow cytometry and fluorescently labeled antibodies. Results are mean ± SEM n=7 mice per group for Large and small peritoneal macrophages n= 7 and splenic macrophages n=4 mice per group. * P < 0.05; ** P <0.01 vs WT vehicle group; # P < 0.05; ** P <0.01 vs ALOX15 vehicle group.
